# Succinate promotes pulmonary fibrosis through GPR91 and predicts death in idiopathic pulmonary fibrosis

**DOI:** 10.1038/s41598-024-64844-5

**Published:** 2024-06-22

**Authors:** Yijun He, Yuanyuan Han, Lijun Zou, Tingting Yao, Yan Zhang, Xin Lv, Mao Jiang, Lingzhi Long, Mengyu Li, Xiaoyun Cheng, Guoliang Jiang, Zhangzhe Peng, Lijian Tao, Jie Meng, Wei Xie

**Affiliations:** 1 Department of Pulmonary and Critical Care Medicine, Third Xiangya Hospital, Central South University, No.138 Tongzipo Road, Changsha, 410013 Hunan China; 2 Hunan Key Laboratory of Organ Fibrosis, Changsha, China; 3 Department of Nephrology, Xiangya Hospital, Central South University, Changsha, China; 4 Department of Pulmonary and Critical Care Medicine, Xiangya Hospital, Central South University, Changsha, China; 5 National International Collaborative Research Center for Medical Metabolomics, Changsha, China; 6 Department of Cardiology, Xiangya Hospital, Central South University, Changsha, China

**Keywords:** Idiopathic pulmonary fibrosis, Energy metabolism, Succinate, GPR91, Cell biology, Diseases, Medical research, Pathogenesis, Risk factors

## Abstract

Idiopathic pulmonary fibrosis (IPF) is believed to be associated with a notable disruption of cellular energy metabolism. By detecting the changes of energy metabolites in the serum of patients with pulmonary fibrosis, we aimed to investigate the diagnostic and prognostic value of energy metabolites in IPF, and further elucidated the mechanism of their involvement in pulmonary fibrosis. Through metabolomics research, it was discovered that the TCA cycle intermediates changed dramatically in IPF patients. In another validation cohort of 55 patients with IPF compared to 19 healthy controls, it was found that succinate, an intermediate product of TCA cycle, has diagnostic and prognostic value in IPF. The cut-off levels of serum succinate were 98.36 μM for distinguishing IPF from healthy controls (sensitivity, 83.64%; specificity, 63.16%; likelihood ratio, 2.27, respectively). Moreover, a high serum succinate level was independently associated with higher rates of disease progression (OR 13.087, 95%CI (2.819–60.761)) and mortality (HR 3.418, 95% CI (1.308–8.927)). In addition, accumulation of succinate and increased expression of the succinate receptor GPR91 were found in both IPF patients and BLM mouse models of pulmonary fibrosis. Reducing succinate accumulation in BLM mice alleviated pulmonary fibrosis and 21d mortality, while exogenous administration of succinate can aggravate pulmonary fibrosis in BLM mice. Furthermore, GPR91 deficiency protected against lung fibrosis caused by BLM. In vitro, succinate promoted the activation of lung fibroblasts by activating ERK pathway through GPR91. In summary, succinate is a promising biomarker for diagnosis and prognosis of IPF. The accumulation of succinate may promote fibroblast activation through GPR91 and pulmonary fibrosis.

## Introduction

Idiopathic pulmonary fibrosis (IPF) is a chronic, progressive, fibrotic interstitial lung disease of unknown etiology characterized by formation of myofibroblast (myo-Fbs) foci and excessive deposition of extracellular matrix (ECM) in the lung, leading to median death 2–3 years after diagnosis^1–3^. To date, there are only two FDA-approved anti-fibrosis drugs in IPF, Nidanib and pirfenidone. However, these drugs only delay the decline of lung function^4–7^. There is a tremendous variability in inter-individual disease course. Currently, clinicians chiefly depend on clinical information—history, radiology, lung function examinationand histopathological model for diagnosis and follow-up of IPF^8–10^. There is an urgent need for diagnostic and prognostic biomarkers that can be used for early diagnosis and to identify which patients are predicted to progress rapidly in IPF.

An increasing number of studies have proposed that the energy metabolic pathways to IPF were abnormal^11,12^. Metabolic profiling has affirmed that metabolites of glycolysis, tricarboxylic acid cycle (TCA cycle), fatty acid and arginine metabolism were altered to varying degrees in IPF patients^13^. Additionally, metabolomic profiling of BLM-induced pulmonary fibrosis in mice identified that disturbed energy metabolism was firmly connected with increased collagen synthesis and protein degradation^14^. However, how energy metabolic changes affect the pathogenesis of pulmonary fibrosis in IPF remains unclear. Further research is needed to determine whether energy metabolism can provide biomarkers for diagnosis and prognosis of IPF.

In the present study, we aimed to investigate the associations between key differential energy metabolic small molecules with diagnosis and prognosis of IPF, and the possible mechanism of its involvement in pulmonary fibrosis.

## Materials and methods

### Participants

Firstly, serum samples from patients with IPF and healthy controls were gathered during a similar time span for energy metabolic assessment (IPF serum, n = 10; control serum, n = 6, the characteristics of patients displayed in Supplemental Table 1). Then we measured differential energy metabolism small molecules levels in a larger cohort with 55 patients with IPF and 19 healthy controls by assay kits (sample size calculation displayed in Supplemental Table 2). The baseline characteristics of the validation cohort available were summarized in Table 1. The diagnosis of IPF depended on the international consensus statement by the American Thoracic Society and the European Respiratory Society through a multidisciplinary discussion of experts in every institution^10^. Fasting blood was collected from all patients in the early morning. Tests of serum were frozen and stored in − 80 °C preceding analysis. The maintained database included clinical, radiological and follow-up information. The GAP indexes and staging system was based on the method previously published^15^. Progressive IPF was defined as ≥ 10% relative decrease in %FVC or death within 1 year after IPF diagnosis^16–18^.

**Table 1:** Baseline demographic and clinical characteristics of patients with healthy controls and IPF in validation cohort.

Characteristic	Healthy control (n = 19)	IPF patients (n = 55)		*p*-Value
		Stable IPF (n = 26)	Progressive IPF (n = 29)	
Age, yr, mean (SD)	62.73 (6.48)	61.31 (6.57)	64.55 (9.52)	0.3138
Gender, male (%)	13 (68.42)	21 (72.41)	18 (69.23)	0.9493
BMI, kg/m^2^, mean (SD)	25.30 (1.10)	25.47 (3.33)	23.21 (3.48)	0.0213
Smoke (%)	10 (52.63)	14 (53.85)	17 (58.62)	0.9163
PaO_2_, mmHg, mean (SD)	–	82.31 (8.09)	76.76 (11.11)	0.0597
%FVC, mean (SD)	–	88.38 (8.19)	80.41 (14.93)	0.0028
%DLCO, mean (SD)	–	55.23 (11.49)	52.36 (10.71)	0.4336
GAP stage (I/II/III)	–	18/7/1	9/15/5	0.0040

Data are presented as mean (SD) or number (%).

yr, year; BMI, body mass index; PaO_2_, arterial oxygen pressure; %FVC, percent predicted FVC; %DLCO, percent predicted diffusing capacity of the lung for carbon monoxide; GAP, gender-age-physiology index.

Patients with IPF were monitored at our outpatient clinic for at least 24 months/until death. Surgical tissue samples of lung from IPF patients were collected from Second Xiangya Hospital of Central South University, and normal nodule-adjacent tissues from pulmonary lobectomy specimens were collected as the non-IPF control group (non-IPF lung samples, n = 6; IPF lung samples, n = 6). The Medical Ethics Committee at Xiangya Hospital of Central South University has approved this study (licence number: 201812184).

### Central carbon metabolomics analysis

Around 100 μl of serum per sample was processed through central carbon metabolomics analysis, which consists of 65 metabolites in six major metabolic pathways^19^. The liquid chromatography-mass spectrometry (LC–MS) condition was based on the method previously published by Kutzner^20^. In brief, after the sample was thawed, then vortexd for 30 s, centrifuged at 3000 r/min for 5 min at 4 °C. 50 μl of the sample was transferred to a centrifuge tube, and mixed with 150 μl of methanol, vortexed for 3 min, centrifuged at 12,000 r/min for 5 min at 4 °C. The 150 μl supernatant was transferred into a new centrifuge tube and placed in the refrigerator − 20 °C for 30 min, centrifuged at 15,000 r/min for 20 min at 4 °C. Afterwards, 140 μl of supernatant was adopted for LC–MS analysis. Energy and its metabolites were detected by MetWare (http://www.metware.cn/) according to AB Sciex QTRAP 6500 LC–MS/MS platform. The analytical conditions were as follows, HPLC: column, ACQUITY UPLC BEH Amide (i.d.2.1 × 100 mm, 1.7 μm); solvent system, water 10 mM Ammonium acetate and 0.3% Ammonium hydroxide (A), acetonitrile with 90% ACN/water(V/V) (B). The gradient was started at 95% B (0–1.2 min), decreased to 70% B (8 min), 50% B (9–11 min), and finally returned to 95% B (11.1–15 min). The flow rate was 0.4 ml/min, the temperature was 40 °C, and the volume of injection was 2 μl.

### Succinate assay

The serum and lung tissue concentrations of succinate were quantified with the Succinate Colorimetric Analysis Kit (MAK184, sigma) according to the manufacturer’s instructions. Briefly, added 10–50 μl serum samples in duplicate wells of a 96-well plate. Tissue (5 mg) was rapidly homogenized on ice in 100 μl of ice-cold Succinate Assay Buffer. Centrifugation with 10,000 × g for 5 min to remove insoluble material. Collected the supernatant, and achieved a final volume of 50 μl with the Succinate Assay Buffer. Then added 50 μl of the appropriate Reaction Mix to each of the wells. Mixed well and incubation the reaction for 30 min at 37 °C. Measured the absorbance at 450 nm (A450). Concentration of succinate was calculated from the standard curve.

### Pyruvic acid assay

Pyruvic acid level in serum was quantified with the Pyruvic Acid Assay Kit (BC2205, Solarbio, China) according to the manufacturer’s instructions. Briefly, 100 μl serum samples were adding to 1 ml pyruvic acid extract buffer. Mixing tubes and incubating the reaction on ice for 30 min. Centrifuging at 8000 × g for 10 min to remove insoluble material. Collecting the supernatant, adding 75 μl samples into duplicate wells of a 96-well plate. Then adding 25 μl reaction reagent 1 to each of the wells. Letting stand for 2 min and adding 125 μl reaction reagent 2 to each of the wells. Mixing well and measuring the absorbance at 520 nm (A520). Concentration of pyruvic acid was calculated from the standard curve.

### Citric acid assay

Citric acid level in serum was quantified with the Citric Acid Assay Kit (BC2155, Solarbio, China) according to the manufacturer’s instructions. Briefly, taking 0.1 ml serum and adding 0.9 ml reaction reagent 1. Mixing tubes and centrifuging at 11,000 × g for 10 min at 4 °C. Collecting the supernatant, adding 20 μl samples into duplicate wells of a 96-well plate. Then adding 140 μl reaction reagent 1 to each of the wells. Then adding 20 μl of reaction reagent 4 and 5 to each of the wells, respectively. Mixing well and letting stand for 30 min at room temperature, measuring the absorbance at 545 nm (A545). Concentration of citric acid was calculated by comparison with standard reagents.

### Animal studies

All the animal studies were approved by the Laboratory Animal Welfare Ethics Committee at Central South University (permit number: 2020sydw0474). All methods were performed in accordance with the relevant guidelines and regulations of Institutional Animal Care and Use Committee (IACUC) of Central South University (Approved protocol No. FORM-AD-AQGF-02). Eight weeks old C57BL/6 mice were purchased from Silaike Company (Changsha, China), housed at Central South University, and provided with ad libitum access to food and water. Succinate selectively binds to its receptor GPR91, also called succinate receptor 1^21^. GPR91-defect mice (GPR91−/− mice) or wild type mice (WT mice) (8 weeks old, 20–25 g weight, kindly provided by Model Animal Research Center of Nanjing University, China) were bred with a C57BL/6 background.

### Establishment of the pulmonary fibrosis mouse model

Two animal experiments were conducted: (1) to observe the changes of succinate level and the expression of GPR91 in BLM induced pulmonary fibrosis mice (C57BL/6 mice) , as well as the effects of administrated with exogenous succinate and dimethyl malonate (DMM) in BLM-challenged mice, DMM is a potent competitive inhibitor of SDH, which could inhibit succinate accumulation in several conditions^22–24^; (2) to observe the effects of GPR91 deficiency in BLM-induced pulmonary fibrosis in mice (C57BL/6 background). In the first experiment, all the 60 mice were randomly divided into the following five groups with twelve mice per group: saline group (Control group), BLM model group (BLM group), succinate-treated (70 mg/kg/d) group (BLM + Suc group) and DMM-treated (150 mg/kg/d) group (BLM + DMM group). In the second experiment, all the 48 mice were randomly divided into WT control group, WT BLM group, GPR91 deficiency control group (GPR91−/− control group) and GPR91 deficiency BLM group (GPR91−/− BLM group). In all model groups, mice were trans-tracheal instilled with 5 mg/kg BLM (Nippon Kayaku, Japan) at day 0, and the experimental control mice received phosphate buffered saline (PBS). Succinate and DMM (Sigma, USA) were administered by intra-peritoneally injection in 100 ul saline daily for 21 days respectively. All mice were sacrificed by deep anesthesia and a high concentration of CO2 asphyxia method at day 21. Lung tissues, serum and bronchoalveolar lavage fluids (BALFs) were collected and stored for subsequent experiments. All animal experiments were performed in accordance with ARRIVE guidelines 2.0.

### Histopathology and immunohistochemistry

Lungs were perfused in situ with cold saline before expulsion. The right lungs were snap-frozen in liquid nitrogen for western blot, succinate quantification test, and quantitative reverse transcription PCR (RT-qPCR) analysis. The left lungs were fixed in paraformaldehyde buffer, embedded in paraffin, and sectioned at 4 µm. Hematoxylin and Eosin (H&E) staining and Masson’s trichrome staining were performed following the manufacturers’ protocols. Alveolitis score^25^ and Ashcroft score^26^ were respectively applied to assess the inflammation and fibrosis of the lungs. For immunohistochemistry, the sections were rehydrated via gradient ethanol, treated with unmasking solution for antigen retrieval, and incubated with primary antibodies at 4 °C overnight. Slides were further stained with secondary antibodies and DAB the following day. Images were obtained at Central South University on a Nikon microscope, and quantified using Image-Pro Plus 7.0.

### Immunofluorescence staining

Tissue paraffin sections were placed in the 65 °C baked for 2 h, dewaxed and hydrated to repair the antigen. Tissue sections were permeabilized with 0.2% Triton X-100 and subsequently blocked, and primary antibodies were diluted 1:100 and incubated overnight at 4 °C. The specimens were incubated with secondary antibody for 45 min protected from light. After DAPI re-staining of cell nuclei, the specimens were microscopically photographed.

### Cell culture and treatment

Normal human lung fibroblasts (NHLFs) were primarily cultured from normal human lungs as previously reported^27^. IPF human lung fibroblasts (HLF) were extracted in the same way from fibrotic lung tissue of IPF patients undergoing lung transplantation. Cells were grown in DMEM medium (Gibco, USA) supplemented with 10% FBS (Gibco, USA) and 1% Penicillin–Streptomycin Liquid (Gibco, USA), and maintained at 37 °C with 5% CO_2_. When the cells grew to 70–80% of confluence, they were incubated with serum-free medium overnight. TGF-β (Peprotech, USA), succinate (Sigma Aldrich, USA) was filtered with 0.22 µm filters before use. NHLFs were incubated with TGF-β for 24 h to construct a fibrosis cell model, and DMM or succinate treatment was given 2 h before TGF-β. GPR91 inhibitors (NF-56-EJ40, MCE, USA) or ERK inhibitors (FR180204, MCE, USA) were administered 4 h before TGF-β or 2 h before succinate. Each experiment was replicated for at least three times.

### Detection in BALFs

BALFs were collected by intratracheal instillation of 1.5 ml (0.5 ml each time, repeat 3 times) of sterile saline solution with gentle aspiration. The recovery of the lavage fluids ranged from 1.2 to 1.4 ml. The protein in BALFs was measured using the BCA Protein Assay Kit (Thermo Fisher Scientific, USA).

### Western blotting

The total protein from lung tissues or cells was extracted using RIPA buffer containing protease inhibitor. Collected lysates were boiled at 95 °C for 5 min, separated on 8–12% SDS-PAGE gels, and transferred to PVDF membranes. Membranes were blocked and then incubated overnight at 4 °C with primary antibodies from the following sources: FN (Abcam, USA), α-SMA (Sigma, USA), p-ERK1/2 (CST, USA), ERK1/2 (CST, USA), p-38 (CST, USA), p-p38 (CST, USA), HIF-1α (Proteintech, USA), α-tubulin (Proteintech, USA), GAPDH (Proteintech, USA), and GPR91 (Novus Biologicals, USA). The secondary antibodies of anti-mouse IgG HRP-linked antibody (#7076) and anti-rabbit IgG HRP-linked antibody (#7074) were purchased from Cell Signaling Technology. The bands were visualized with ECL detection reagents (Thermo Fisher Scientific, USA) and quantified using ImageJ software.

### RNA extraction and real-time PCR quantitation

Total RNA was extracted from lung tissues with Trizol reagent (Thermo Fisher Scientific, USA) according to the manufacturer’s instructions. Reverse transcription reactions were carried out with 1 μg of total RNA using a cDNA synthesis kit (Thermo Fisher Scientific, USA). The synthesized cDNA was further used for PCR where β-actin was used as loading control. SYBR Green gene expression assay (Thermo Fisher Scientific, USA) was performed in a CFX96 Real-Time System (Bio-Rad Laboratories, USA) to measure the mRNA expression. The specific primers were designed from the GenBank sequences and synthesized by Sangon Biotec (Shanghai, China). The forward primer of human β-actin was 5′-GGCCAACCGTGAAAAGATGA-3′, and the reverse primer was 5′-GACCAGAGGCATACAGGGACAA-3′. The forward primer of mouse β-actin was 5′-CATTGCTGACAGGATGCAGAAGG-3′, and the reverse primer was 5′-TGCTGGAAGGTGGACAGTGAGG-3′. The forward primer of mouse GPR91 was 5′-GACAGAAGCCGACAGCAGAATG-3′, and the reverse primer was 5′-GCAGAAGAGGTAGCCAAACACC-3′. The forward primer of human GPR91 was 5′-GGAGACCCCAACTACAACCTC-3′, and the reverse primer was 5′-AGCAACCTGCCTATTCCTCTG-3′.

### Statistical analysis

Raw LC–MS data were processed, normalized (quantile), log2 transformed and auto scaled. Principal component analysis (PCA), hierarchical clustering and statistical t-test were conducted on normalized data by R software (R version 4.1.3), and R package “ggplot2” was used to visualize PCA analysis. Statistically significant by t-test was considered for features with FDR corrected *p* value < 0.05. The hierarchical cluster analysis (HCA) results of samples and identified metabolites were presented as heatmaps with dendrograms carried out by the R package ‘pheatmap’. For HCA, normalized signal intensities of metabolites (unit variance scaling) are visualized as a color spectrum. Identified metabolites were then assigned to their class of compounds as described by the Kyoto Encyclopedia of Genes and Genomes (KEGG) and Human Metabolome Database (HMDB). Subsequently, they were individually mapped onto their major biochemical pathways to holistically visualize the metabolic shift. During this process, the R package “ggplot2” was employed to present the results of KEGG analysis. Continuous and categorical variables were expressed as mean ± SD and number (%), respectively. Comparisons between groups were performed either with a parametric unpaired two-tailed Student’s t-test or non-parametric two-tailed Mann–Whitney test. Comparisons among three or more independent groups were done by one-way analysis of variance (ANOVA) with Tukey’s multiple comparison post-test. The Pearson correlation coefficient was used to analyze the correlation between variables. Receiver operating characteristic (ROC) curve analysis was carried out to identify the optimal cutoff value. The cumulative survival rates were estimated by the Kaplan–Meier method; and the log-rank test was used to assess between-group differences. Logistic regression analysis was used to identify variables that were associated with disease progression. Cox proportional hazards regression analysis with time-dependent covariance was used to identify the prognostic factors. In all data analysis, *p* < 0.05 was considered significantly different. The analyses were conducted using SPSS 25.0, and GraphPad Prism 8.0.

### Ethics approval

The Medical Ethics Committee of Xiangya Hospital of Central South University approved this study (permit number: 201812184). All patient care and research were conducted in compliance with the Declaration of Helsinki. All patients provided written informed consent prior to inclusion in the study. Participants gave informed consent to participate in the study before taking part.

## Results

### The intermediate products of TCA cycle were related with IPF

**Figure 1:**
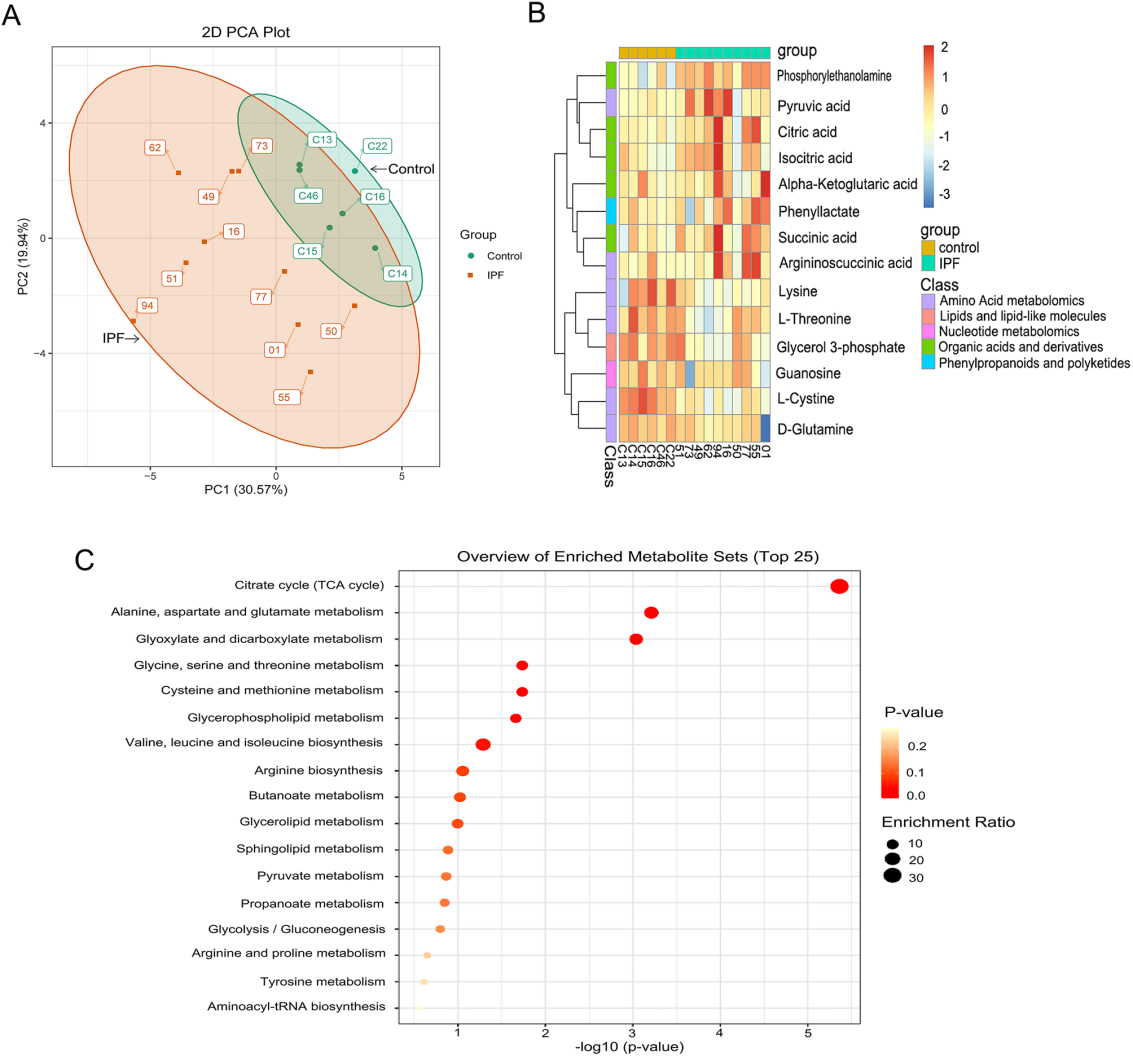
Energy metabonomics profiling of human serum heterogeneity. R software (R version 4.1.3) was used to perform principal component analysis (PCA) and package “ggplot2” was used to visualize the PCA results, which showed clear separation of metabolite profiles between IPF and healthy control (**A**). The hierarchical cluster analysis (HCA) results of the samples and the identified metabolites were presented as heat maps via the R packet “pheatmap”, which showed shows 14 differential metabolites, categorized by IPF and control groups (**B**). KEGG metabolic pathway analysis results of differential metabolites were visualized by R package “ggplot2”, as shown in (**C**).

The LC–MS based metabolic profiling study on levels of metabolites from human serum specimens was outlined in (Supplemental Tables 3 and 4). A total of 57 metabolites were designated by using authentic standards, and 41 metabolites were suitable for statistical analysis. The principal component analysis (PCA) showed clear separation of metabolite profiles between IPF and healthy control (Fig. 1A). We have identified 14 metabolites which were statistically different (FDR-corrected *p* value < 0.05) between the two groups (Fig. 1B), of which 13 metabolites had both HMDB and KEGG identifiers. There were 7 up-regulated and 6 down-regulated metabolites in IPF (Supplemental Table 4). As expected, the intermediate products of the TCA cycle were significantly changed, which indicated the dysfunction of glycolysis and TCA cycle (Fig. 1C). Elevated glycolysis levels produced large amounts of pyruvate. As we observed, pyruvate levels were significantly increased in IPF patients compared to healthy controls (fold change = 2.32, *p* < 0.01). While the intermediate products of TCA cycle such as succinic acid (fold change = 1.38, *p* < 0.05), citric acid (fold change = 1.86, *p* < 0.05) and isocitric acid (fold change = 2.11, *p* < 0.05) all increased in different degrees. However, we didn’t observe significantly changes in fumaric acid (fold change = 0.96, *p* = 0.62), the downstream molecule of succinic acid in the TCA cycle. These results suggested that there was a significantly dysfunction of the TCA cycle in IPF patients.

### The level of succinate was increased in another validation IPF cohort

**Figure 2:**
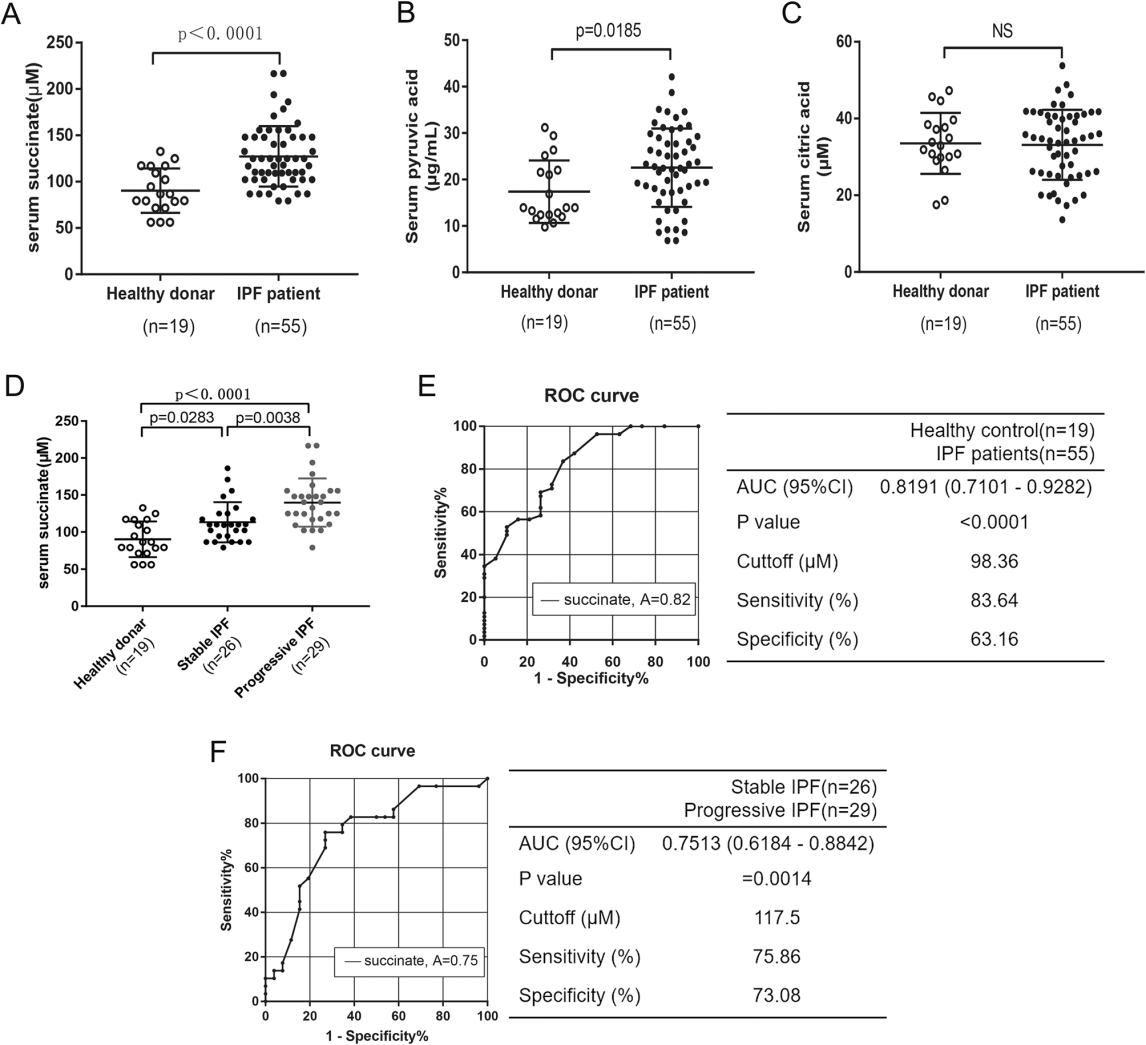
Serum succinate levels increased in IPF patients. (**A**) Graph shows succinate levels in serum from healthy donors (n = 19) and IPF patients (n = 55). Graphs show pyruvic acid levels (**B**) and citric acid levels (**C**) in serum from IPF cohort and healthy controls. (**D**) Graph shows succinate levels in serum from healthy donors and subgroup of IPF cohort (Stable IPF, n = 26; Progressive IPF, n = 29). (**E**) ROC Analysis of serum succinate to distinguish IPF from healthy controls. (**F**) ROC Analysis of serum succinate as a marker in predicting progression of IPF. Abbreviations: AUC, Area Under the Curve; CI, confidence interval.

To determine the clinical significance of intermediate molecules of the TCA cycle in IPF, we measured pyruvic acid (Fig. 2B), citric acid (Fig. 2C) and succinate (Fig. 2A) levels in serum from an IPF cohort and healthy controls. Additionally, succinate may be worth studying in pulmonary fibrosis. In ischemia reperfusion injury, succinate has been identified as an extracellular inflammation signal molecule^28^. As shows in Fig. 2A, the succinate level was significantly higher in the IPF cohort than in the healthy controls (IPF cohort, 127.2 ± 4.398 μM; healthy controls, 90.35 ± 5.509 μM). On the basis of follow-up data, IPF patients were divided into progressive (n = 29) and stable (n = 26) groups. The succinate level was higher in the progressive IPF subgroup than the stable IPF subgroup (Fig. 2D) (progressive IPF, 139.8 ± 6.054 μM; stable IPF, 113.3 ± 5.278 μM). ROC analysis was used to assess serum succinate’s sensitivity and specificity in discriminating IPF from healthy controls (Fig. 2E) and assessing disease progression within cohort of IPF patients (Fig. 2F). IPF was distinguished by serum succinate by an area under the curve of 0.8191 (95% confidence interval (CI) 0.7101 to 0.9282; *p* = 0.0001), while progressive IPF was distinguished by serum succinate by an area under the curve of 0.7513 (95% confidence interval, 0.6184 to 0.8842; *p* = 0.0014). The cut-off levels of serum succinate were 98.36 μM for distinguishing IPF from healthy controls (sensitivity, 83.64%; specificity, 63.16%; likelihood ratio, 2.27, respectively) and 117.5 μM for predicting disease progression (sensitivity, 68.97%; specificity, 73.08%; likelihood ratio, 2.81, respectively), respectively. Based on the Pearson correlation coefficient, we analyzed the relationship between succinate level and clinical parameters (Supplemental Fig. 1), including age, body mass index (BMI), arterial partial pressure of oxygen (PaO_2_), predicted forced vital capacity (%FVC) and predicted diffusing capacity for carbon monoxide (%DLCO). There was no significant correlation between serum succinate and age (r = 0.1014, *p* = 0.4492), BMI (r = − 0.1290, *p* = 0.3480), PaO_2_ (r = 0.0109, *p* = 0.9372), %FVC (r = − 0.0094, *p* = 0.9460), or %DLCO (r = − 0.2295, *p* = 0.0919), respectively.

### Serum succinate predicted clinical outcomes in patients with IPF

**Figure 3:**
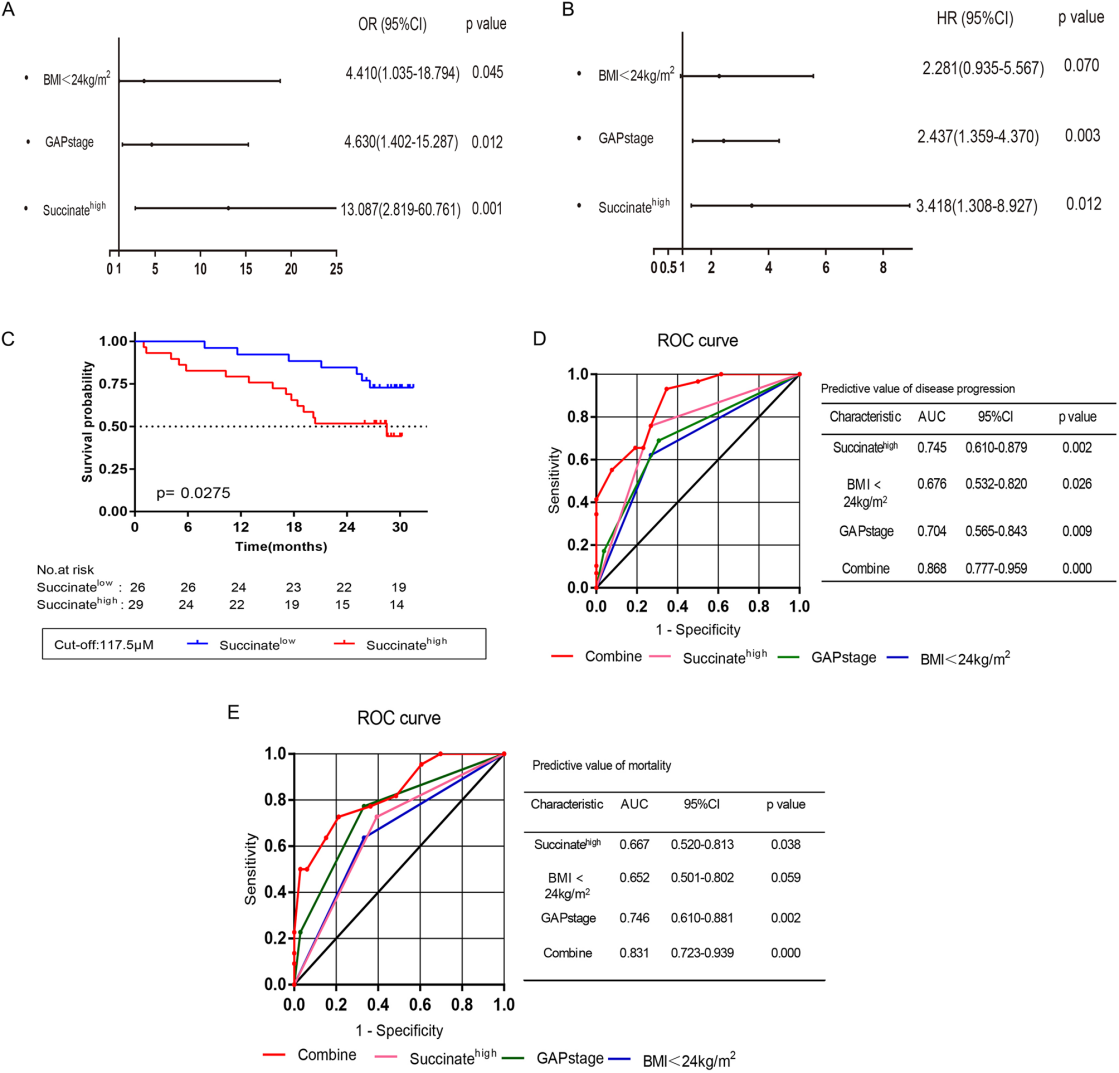
Serum succinate levels predict the prognosis of IPF. (**A**) Alignment diagram of BMI, GAP stage, succinate to predict disease progression. (**B**) Alignment diagram of BMI, GAP stage, and succinate to predict mortality. Kaplan–Meier plot for all-cause mortality shows that the 2-year cumulative survival rate was significantly lower in the Succinate^high^ subgroup than in the Succinate^low^ subgroup (**C**). ROC Analysis of serum succinate, BMI < 24 kg/m^2^, and GAP stage is used to predict disease progression (**D**) and mortality (**E**) in IPF. Abbreviations: OR, odds ratio; HR, hazard ratio; CI, confidence interval; BMI, body mass index; GAP, gender-age-physiology index; Succinate^high^, serum succinate ≥ 117.5 μM; Combine, combine the three indicators (BMI < 24 kg/m^2^, GAP stage and Succinate^high^).

The IPF cohort was classified into succinate^low^ and succinate^high^ subgroups based on an index of serum succinate level (cut-off value was 117.5 μM). All patients enrolled in the study had access to follow-up information 24 to 33 months after enrollment. As described above, disease progression occurred in 29 patients. At the end of follow-up, 33 patients (60.0%) were alive and 22 patients (40.0%) had been dead. The results of the logistic regression analysis of disease progression in IPF patients were presented in Supplemental Table 5. High serum succinate levels independently predicted disease progression in the IPF cohort (succinate^high^ subgroup, odds ratio (OR), 13.087; 95% CI 2.819–60.761; *p* = 0.001). Results of Cox proportional hazard analysis of mortality were presented in Supplemental Table 6. In a multivariate analysis, high serum succinate levels were associated with an increased mortality rate (succinate^high^ subgroup, hazard ratio (HR), 3.418; 95% CI 1.308–8.927; *p* = 0.012). Furthermore, BMI, GAP stage, and succinate all showed predictive value for disease progression and prognosis in the IPF cohort (Fig. 3A,B). Additionally, the cumulative survival rate in the succinate^high^ subgroup was significantly lower than that in the succinate^low^ subgroup (Fig. 3C). Based on ROC analysis, AUC of succinate^high^, GAP stage, BMI > 24 kg/m^2^, and their combined predictions of disease progression were 0.745, 0.704, 0.676, and 0.868 (Fig. 3D), and prediction of all-cause mortality were 0.667, 0.746, 0.652, and 0.831 (Fig. 3E), respectively.

### The succinate-GPR91 *axis* was activated in fibrotic lung tissue

**Figure 4:**
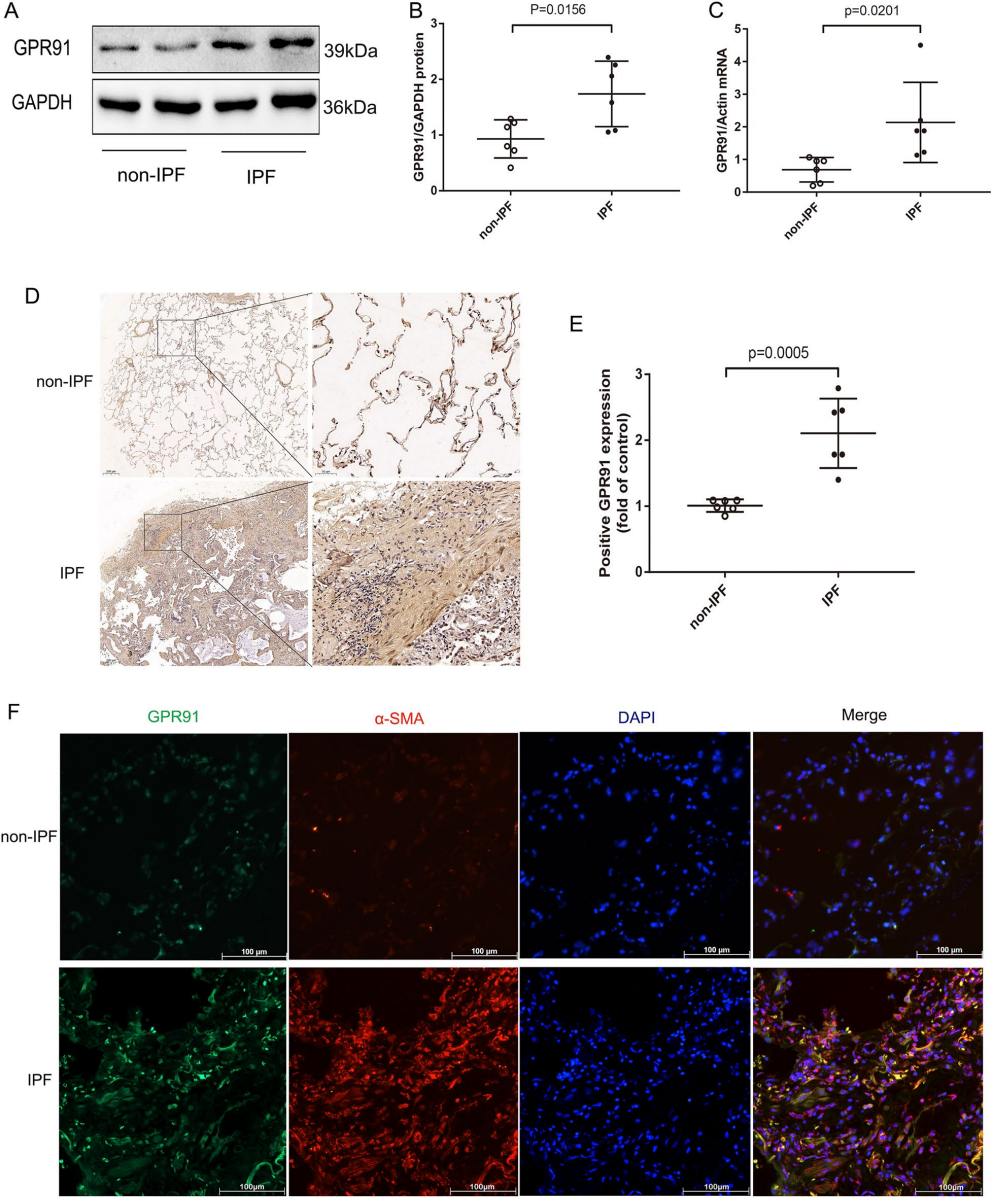
The expression of GPR91 is increased in the lungs of patients with IPF. (**A**) Western blot and (**B**) quantitative analysis of GPR91 in non-IPF patients and IPF patients (n = 6). GPR91 mRNA levels in non-IPF and IPF lung tissues (**C**) (n = 6). Immunohistochemistry for GPR91 in lung tissues from non-IPF and IPF patients (**D** and **E**) (n = 6). In (**D**), the magnifications were × 10 and × 200. Bars in graphs represent mean ± SD. Data were analyzed by two-tailed Student’s t-test. A representative immunofluorescence images (**F**) taken with the fluorescence microscope N2-DM4B showing co-localization of GPR1 in α-SMA positive cells in IPF fibrotic lungs.

**Figure 5:**
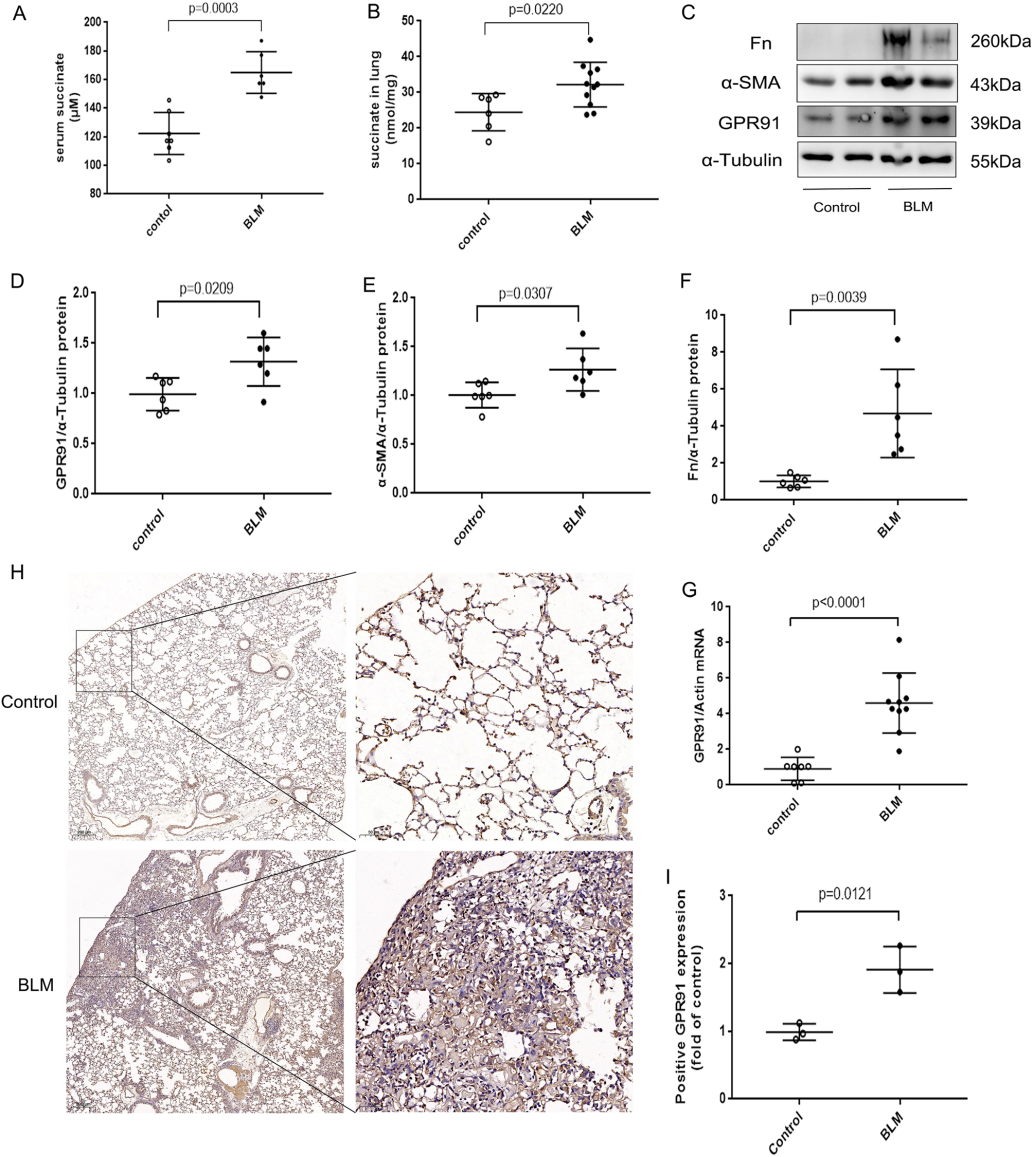
In the BLM-induced pulmonary fibrosis mice model, succinate levels in serum and lung tissue are elevated, and the expression of GPR91 in the lungs is increased. The lungs were collected 21 days after the lungs were challenged with BLM (5 mg/kg, intratracheal instillation) to induce pulmonary fibrosis. Graph shows succinate levels in serum (**A**) and lung (**B**) from control group and BLM group (n ≥ 6). Western blot (**C**) and quantitative analysis of GPR91 (**D**), α-SMA (**E**) and FN (**F**) in the lung tissue from different groups (n ≥ 6). The mRNA levels of GPR91 in lung tissues from different groups (**G**) (n ≥ 6). Representative images (**H**) and quantitative analysis of immunohistochemistry for GPR91 (**I**) in lung tissues from the control group and BLM group (n = 3 per group). The magnifications in (**H**) were × 10 and × 200. All data were presented as mean ± SD. A T-test was used to compare the two groups.

To investigate whether the succinate-GPR91 axis was activated in fibrotic lung tissue, succinate and GPR91 were detected in lung tissue from patients with IPF. The succinate level was significantly increased in fibrotic lung tissues from IPF patients (Supplement Fig. 2A). In lung tissue from IPF patients, GPR91 mRNA and protein levels were higher than those from non-IPF patients (Fig. 4A–C). An immunohistochemical examination of IPF patients’ fibrotic lung lesions revealed increased GPR91 expression (Fig. 4D–E). Immunofluorescence assay showed that GPR91 was co-expressed with α-smooth actin(α-SMA) cells in fibrotic lung tissue from IPF patients (Fig. 4F). The succinate-GPR91 axis activation was also observed in BLM-induced pulmonary fibrosis in mice (Fig. 5). BLM-induced mice had significantly higher serum and lung succinate levels than control mice (Fig. 5A,B). In addition, we found that mice treated with BLM expressed high levels of GPR91 protein and mRNA (Fig. 5C,D,G). Moreover, expressions of GPR91 and fibrosis related markers α-SMA and fibronectin (FN) showed the same trend (Fig. 5C–E). In fibrous lesions of BLM-induced mice, GPR91 was highly expressed by immunohistochemistry (Fig. 5H–I). Accordingly, pulmonary fibrosis was closely associated with activated succinate-GPR91 axis.

### Succinate aggravated BLM-induced mice pulmonary fibrosis through GPR91

**Figure 6:**
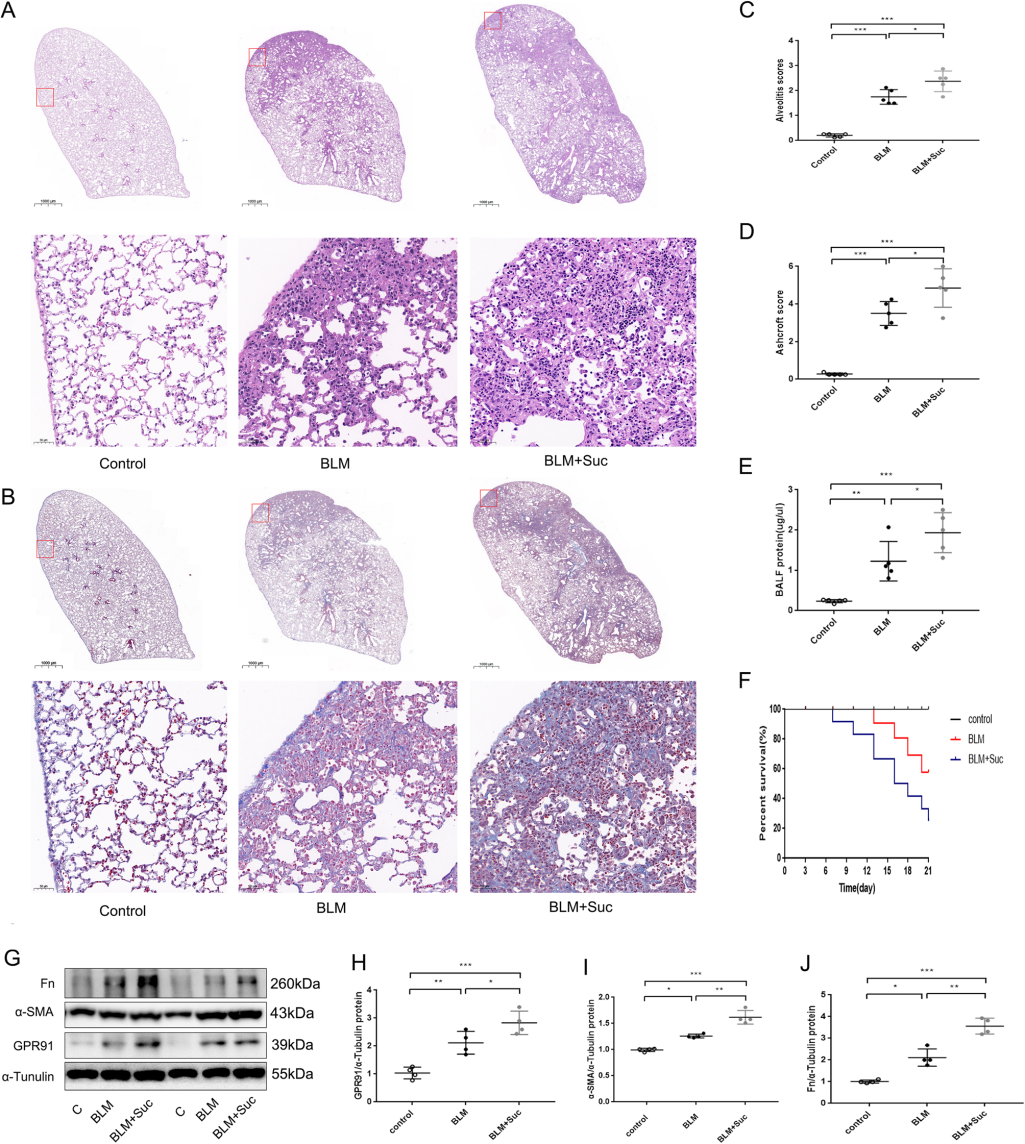
Exogenous succinate exacerbates lung fibrosis and mortality in the BLM-induced pulmonary fibrosis mouse model. As described above, the BLM-induced mouse model is used. In BLM + Suc mice, succinate was injected intraperitoneally for 21 days at 70 mg/kg/d. Representative images showing H&E staining (**A**) and Masson’s trichrome staining (**B**) of lung tissues from different groups of mice. (**C**,**D**): Alveolitis scoring (**C**) and Ashcroft scoring (**D**) for pulmonary damage and fibrosis (n = 5). Different groups’ total protein concentrations in BALF (**E**) (n = 5). A survival analysis of lung fibrosis mice induced by BLM(F). (**G**) Western blot and analysis of GPR91, a-SMA, and FN in the lung tissue (**H**–**J**). Quantification of GPR91, a-SMA, and FN in the lung tissue from different groups (n = 4). (**A**,**B**) at magnifications of × 10 and × 200. All data were presented as mean ± SD. ANOVA was used for comparison in (**C**,**D**). *p < 0.05; **p < 0.01; ***p < 0.001. Abbreviations: BALF, Bronchoalveolar Lavage Fluid; Suc, succinate.

Exogenous succinate administration significantly increased mortality (Fig. 6F) and BLM-induced pulmonary fibrosis in mice. In BLM-induced mice, succinate exacerbated inflammatory cell infiltration, structure destruction, and collagen deposition as revealed by H&E dye and Masson’s trichrome staining (Fig. 6A,B). In BLM + Suc group, both Alveolitis and Ashcroft scores were significantly higher than in BLM group (Fig. 6C,D). The total protein in BALF of mice induced by BLM was also increased by succinate (Fig. 6E). Western blots further confirmed that succinate promoted the expression of GPR91 and fibrosis-related markers of α-SMA and FN (Fig. 6G–J). Through GPR91, succinate might aggravate BLM-induced mice pulmonary fibrosis.

### Inhibiting succinate accumulation alleviated BLM-induced mice pulmonary fibrosis

**Figure 7:**
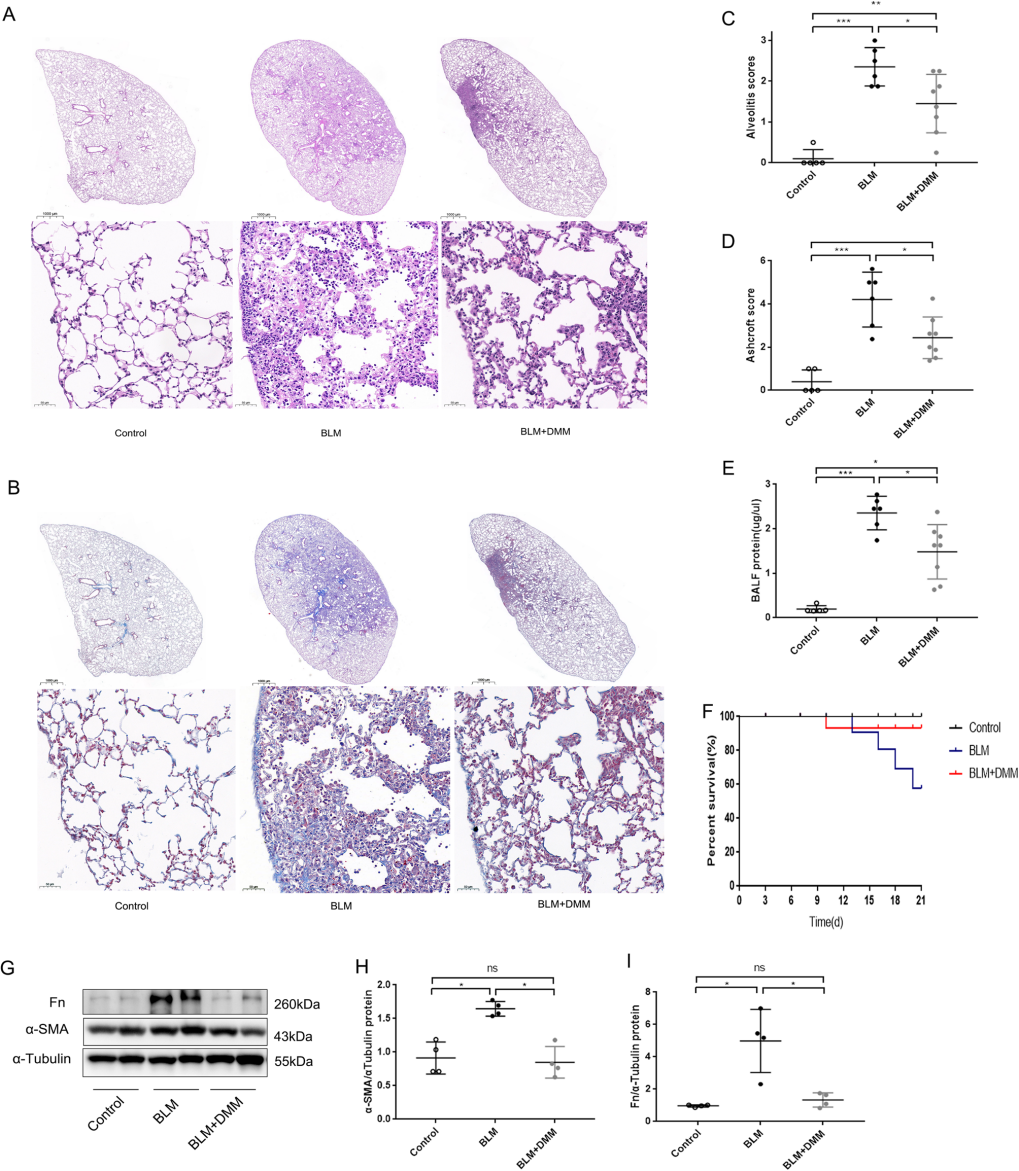
In the BLM induced pulmonary fibrosis mouse model, DMM inhibited succinate accumulation and reduced lung fibrosis and mortality. BLM-induced mouse model is as described above. BLM + DMM mice were treated with DMM (150 mg/kg/d) by intraperitoneal injection for 21 days. A and B: Representative images of H and E staining (**A**) and Masson’s trichrome staining (**B**) of lung tissues from different mouse groups. For different groups, the Alveolitis score (**C**) and Ashcroft score (**D**) are shown(n ≥ 6). Total protein concentration in BALF (**E**) of different groups (n = 5). Western blotting (**F**) and quantitative analysis of α-SMA (**G**) and FN (**H**) in mice lung tissues (n = 4). The survival rate of BLM-induced lung fibrosis mice was analyzed. In (**A**) and (**B**), magnifications were 10 and 200, respectively. All data were presented as mean ± SD. *p < 0.05; **p < 0.01; ***p < 0.001. Abbreviations: BALF, Bronchoalveolar Lavage Fluid; DMM, dimethyl malonate.

To further verify that succinate is a key regulator of pulmonary fibrosis, DMM was used to investigate whether inhibiting succinate accumulation improved pulmonary fibrosis. There was a significant decrease in succinate levels in serum and lung tissues in the BLM + DMM group compared to the BLM model group (Supplement Fig. 2B,C). BLM-induced mice exhibited extensive distortion, massive infiltration of inflammatory cells, and excessive collagen deposition, which were attenuated by DMM treatment (Fig. 7A,B). In addition, the Alveolitis scores (Fig. 7C) and Ashcroft scores (Fig. 7D) confirmed these findings. DMM also reduced BLM-induced protein elevations in BALFs (Fig. 7E). Western blot further verified that DMM treatment reduced α-SMA and FN expression in lung tissue of BLM induced mice (Fig. 7F–H). Survival analysis revealed that DMM significantly increased survival rates for mice challenged with BLM (Fig. 7I). In summary, these findings suggest that inhibiting succinate accumulation ameliorates BLM-induced pulmonary fibrosis.

### GPR91 deficiency protected against BLM-induced mice pulmonary fibrosis

**Figure 8:**
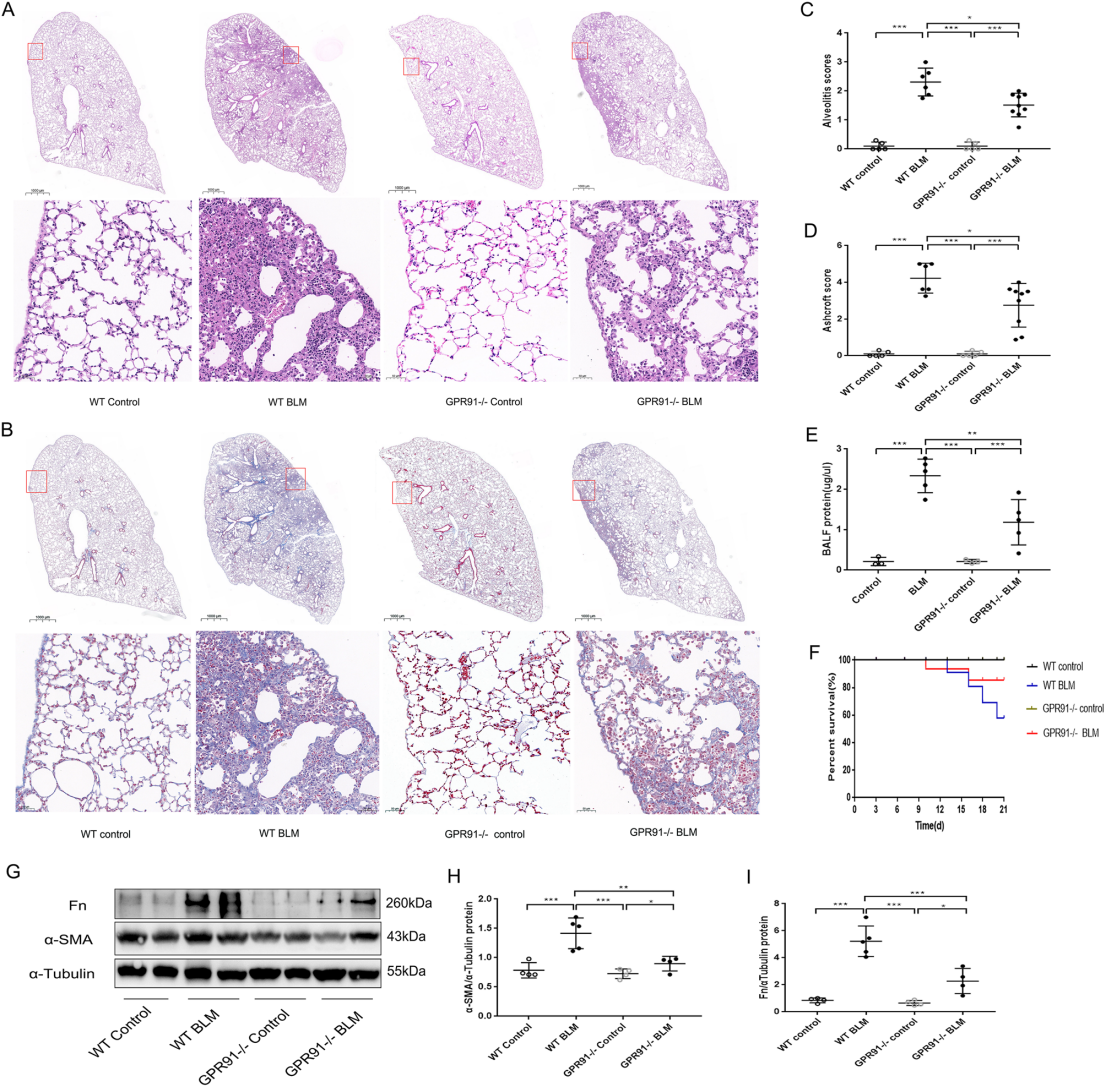
GPR91 deficiency protects mice from pulmonary fibrosis and mortality caused by BLM. BLM (5 mg/kg, intratracheal instillation) induced pulmonary fibrosis in WT mice and GPR91−/− mice. A 21-day period after bleomycin challenge was used to collect the lungs. A and B: Representative images of H&E staining (**A**) and Masson’s trichrome (**B**) of lung tissue from WT and GPR91−/− mice in the control and BLM 21 days groups. Alveolitis scoring (**C**) and Ashcroft scoring (**D**) of different groups (n ≥ 5). Protein concentration in BALF (**E**) of different groups (n = 5). An analysis of the survival rate (**F**) of lung fibrosis mice induced by BLM. Western blotting (**G**) and quantitative analysis of α-SMA (**H**) and FN (**I**) in mice lung tissues (n ≥ 4). The magnification in (**A**) and (**B**) were × 10 and × 200. All data were presented as mean ± SD. *p < 0.05; **p < 0.01; ***p < 0.001.

Moreover, we confirmed that GPR91 plays a role in the pathogenesis of pulmonary fibrosis. We established a BLM-induced pulmonary fibrosis model in WT mice and GPR91−/− mice. H&E staining and Masson’s staining of GPR91−/− mice treated with BLM showed significantly reduced pulmonary alveolitis and collagen deposition compared to BLM-induced WT mice (Fig. 8A–D). As shown in Fig. 8E, GPR91 deficiency ameliorates lung injury after BLM treatment. Figure 8F shows that mice survival rate was markedly increased by GPR91 deficiency after BLM induction. Furthermore, GPR91 deficiency decreased levels of α-SMA and FN in BLM-induced mice (Fig. 8G–I). It was demonstrated that mice with GPR91 deficiency were protected from pulmonary fibrosis induced by BLM.

### Succinate regulated pulmonary fibroblast activation through GPR91

**Figure 9:**
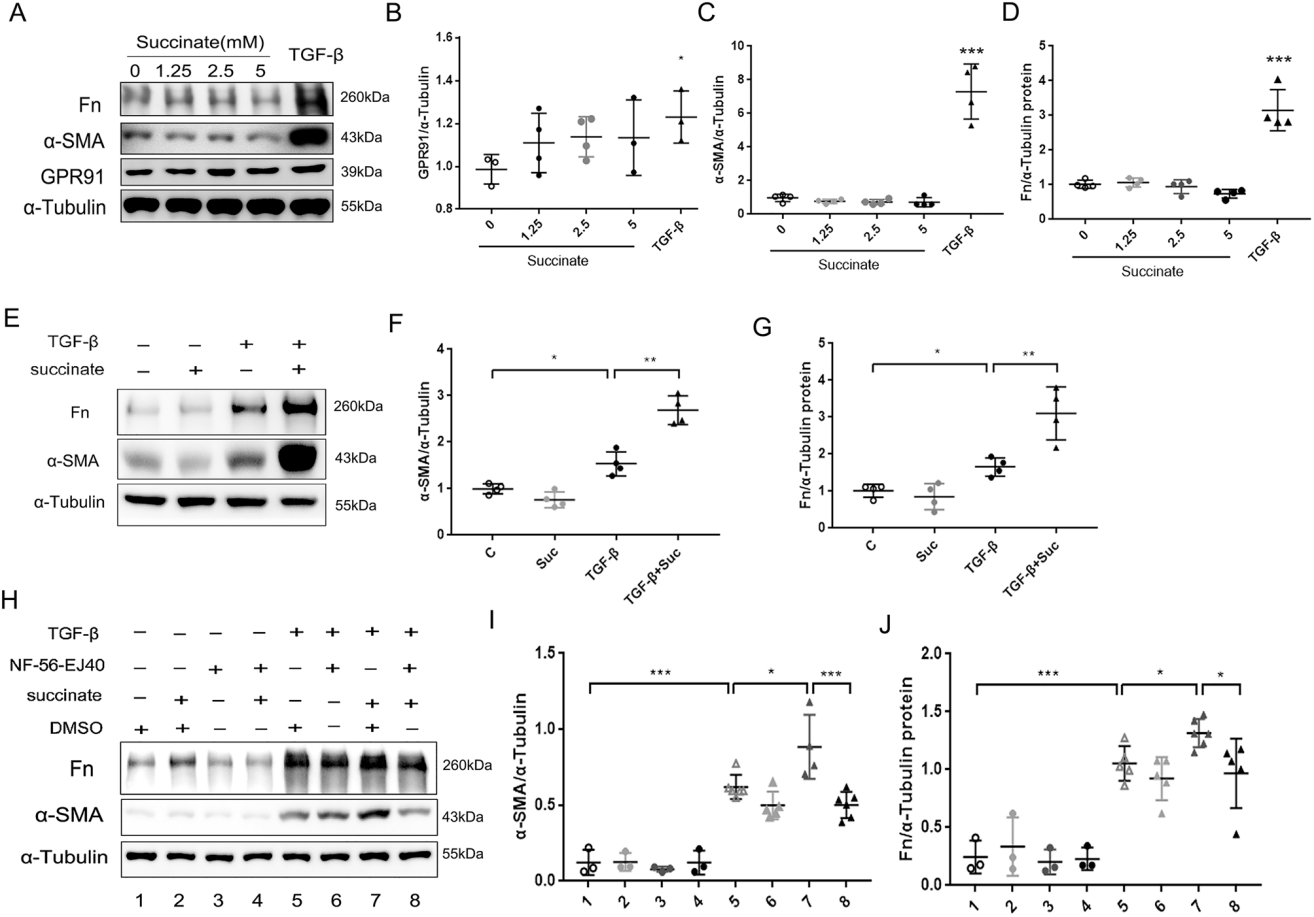
Succinate regulates pulmonary fibroblast activation through GPR91. Western blotting (**A**) and quantitative analysis of GPR91 (**B**), α-SMA (**C**) and FN (**D**) in normal human lung fibroblasts (NHLFs) treated with various concentrations succinate or TGF-β for 24 h. Western blotting (**E**) and quantitative analysis of α-SMA (**F**) and FN (**G**) in NHLFs treated with TGF-β with/without succinate (2.5 mM) pretreatment 2 h. Western blotting (**H**) and quantitative analysis of α-SMA (**I**) and FN (**J**) in NHLFs treated pretreated with NF-56-EJ40 for 2 h and treated with TGF-β with/without succinate (2.5 mM). All experiments were conducted with more than three independent replications. Data were presented as mean ± SD. ANOVA was used for comparison in different groups. *p < 0.05; **p < 0.01; ***p < 0.001.

**Figure 10:**
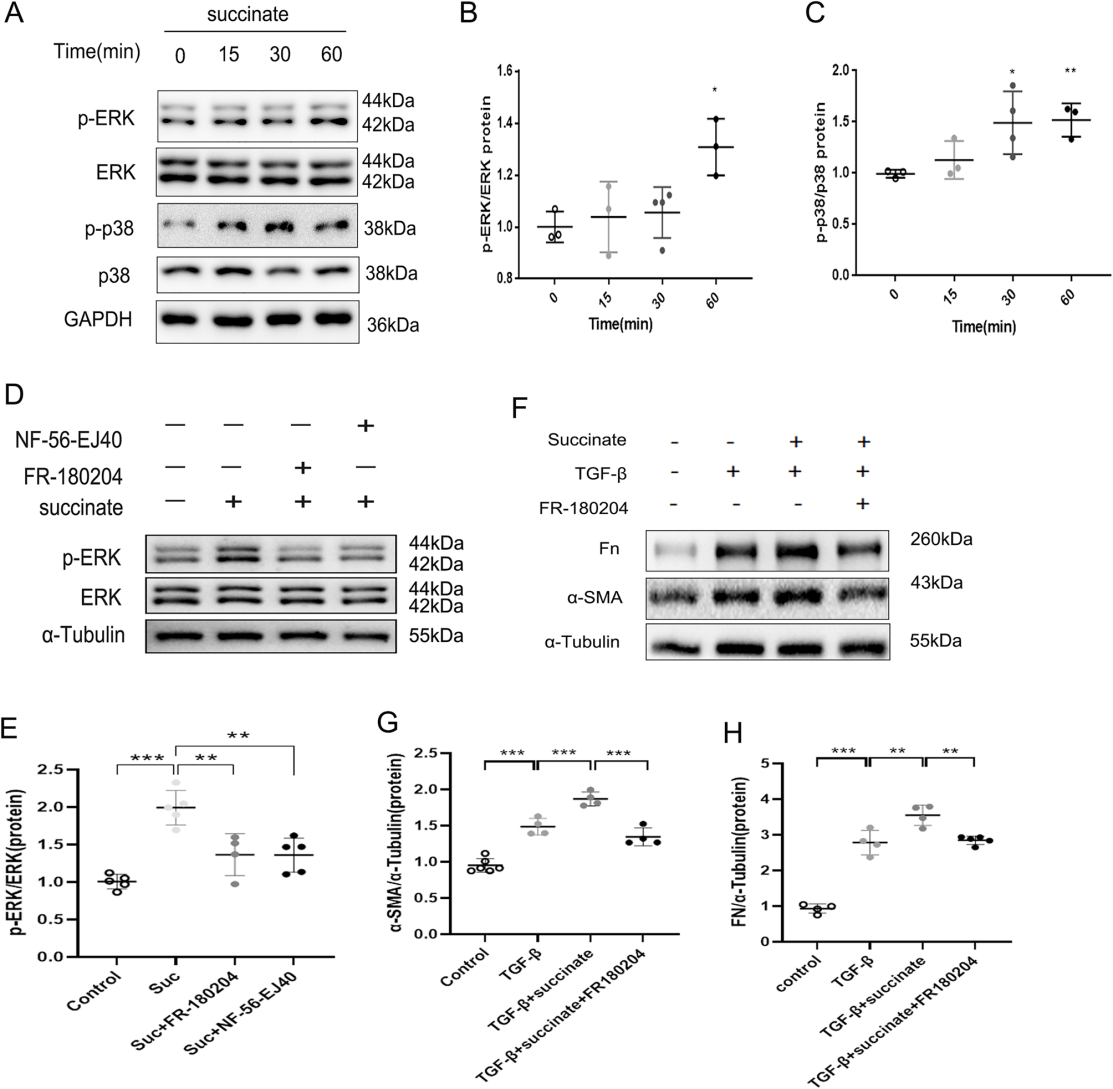
Succinate regulates pulmonary fibroblast activation through ERK phosphorylation. NHLFs treated with succinate at different times were subjected to Western blotting (**A**) and quantitative analysis of p-ERK (**B**) and p-p38 (**C**). Western blotting (**D**) and quantitative analysis of p-ERK (**E**) in NHLFs treated pretreated with NF-56-EJ40 and FR-180204 2 h with/without succinate (2.5 mM). Western blotting (**F**) and quantitative analysis of α-SMA (**G**) and FN (**H**) in NHLFs treated pretreated with FR-180204 for 2 h and treated TGF-β with/without succinate (2.5 mM). All experiments were conducted with more than three independent replications. Data were presented as mean ± SD. ANOVA was used for comparison in different groups. *p < 0.05; **p < 0.01; ***p < 0.001.

In pulmonary fibrosis, fibroblasts maintain stromal homeostasis by integrating signals from the ECM that influence their function and fate^3,29^. In the GSE17978 database(https://www.ncbi.nlm.nih.gov/geo/), we found that the mRNA expression of GPR91 in IPF fibroblasts was higher than that of the normal control, and the expression of GPR91 was positively correlated with α-SMA, FN and COL1 expressions (Supplemental Fig. 3A,B). At the same time, the protein expression of GPR91 in the primary lung fibroblasts of IPF was higher than that of primary normal human lung fibroblasts (NHLFs) (Supplemental Fig. 3C–F). In order to confirm succinate’s ability to control fibrosis in vitro, NHLFs were chosen for testing. As shown in Fig. 9A,C,D, succinate alone did not induce elevated levels of fibrotic markers in NHLFs. Nevertheless, succinate promoted TGF-β induced normal lung fibroblasts activation (Fig. 9E–G). In addition, activated NHLFs expressed significantly higher levels of GPR91 (Fig. 9A,B). Furthermore, NF-56-EJ40 (a highly selective GPR91 antagonist)^30^ alleviated the activation of NHLFs treated with TGF-β or TGF-β plus succinate (Fig. 9H–J). Activation of GPR91 can induce the activation of the MAPK pathway in several cell lines^31–33^. NHLFs treated with succinate showed increased phosphorylation of ERK-1/2 and p38 in comparison with vehicle-treated NHLFs (Fig. 10A–C). GPR91 antagonist inhibits the ERK-1/2 phosphorylation of NHLF induced by succinate (Fig. 10D–C). It was further found that ERK inhibitor FR180204 could reduce α-SMA and FN expression of NHLFs promoted by succinate (Fig. 10F–H). As a result of these data, succinate can influence the activation of normal lung fibroblasts by regulating ERK phosphorylation through GPR91.

## Discussion

Metabolic alteration is increasingly recognized as a pathogenic process affecting fibrosis in many organs, such as the heart, liver, and kidneys^22,31,34^. However, how energy metabolic changes affect the pathogenesis of pulmonary fibrosis remains unclear. In this study, we found significant differences in serum energy metabolism molecules among IPF patients compared to healthy controls, especially succinate, an intermediate of the TCA cycle. As far as we know, this was the first study to determine the serum succinate as a biomarker to identify patients with IPF and predict disease prognosis.

In patients with IPF, energy metabolism disorders were deemed to contribute to the pathogenesis of the disease^35^. The BLM-induced pulmonary fibrosis mouse model also showed elevated glycolysis and disrupted TCA cycle^13,14,36^. TCA cycle is responsible for maintaining energy homeostasis and cell metabolism^37^. Through metabonomics analysis of serum TCA cycle substances in patients with IPF, significant changes were found in pyruvate, isocitrate, succinate, and citric acid levels. This is consistent with the results reported by Zhao et al. in the metabolomics detection of IPF lung tissue^38^ Further, we found that succinate distinguished IPF from healthy controls better than pyruvate and citric acid. Succinate has been identified as an extracellular signaling molecule, participating in pathophysiological processes such as local stress, ischemia and hypoxia^22,39^. In the validation IPF cohort, serum succinate levels were significantly higher than in healthy controls. The cutoff value of serum succinate 98.36 μM was useful to distinguish IPF from normal people (sensitivity, 83.64%; specificity, 63.16%). Patients with IPF who had elevated succinate levels had an increased risk of disease progression and all-cause mortality. In addition, we concluded that the combination of serum succinate, GAP stage, and BMI models could improve prognostic classification in patients with IPF. In terms of predicting IPF outcome, the GAP model and BMI have proven useful^15,40^. High serum succinate levels independently predicted disease progression (with OR 13.087; 95% CI 2.819–60.761; *p* = 0.001; AUC, 0.745) and mortality (with HR 3.418; 95% CI 1.308–8.927; *p* = 0.012; AUC, 0.667). The combination of succinate, GAP stage and BMI can improve the prediction efficiency. While the use of succinate alone was significant for the prediction of IPF and progression, the specificity was relatively low. There may be a variety of factors. In one sense, succinate accumulation reflects energy metabolic disorder. Under pathological conditions, succinate accumulates in a variety of cells^22,23,28^. Aside from that, IPF is a heterogeneous disease, and the course of the disease varies considerably among individuals, making it relatively difficult to predict prognoses. Due to the limited sample size of our cohort, a cohort with a larger sample size may be required for further validation. Overall, we believed that succinate was a promising biomarker for predicting disease progression and mortality in patients with IPF.

We further detected succinate accumulation in fibrotic lung tissue from IPF patients in this study. Similarity to the clinical results above, we found that serum and lung succinate levels increased in BLM-induced mice. A previous study showed that accumulated succinate in mitochondria was transported to the cytosol and then released into the extracellular space^28^. It is unclear whether extracellular succinate promotes pulmonary fibrosis. Our findings in this study indicated that exogenous succinate administered to BLM-induced mice aggravated their lung fibrosis as well as increased their mortality. In contrast, DMM administration to BLM-induced mice reduced succinate concentration, alleviated lung fibrosis and reduced mortality. Succinate signaling may be a therapeutic target for pulmonary fibrosis based on these effects. In vitro experiments, we found succinate accumulation in NHLFs during TGFβ-induced activation, and DMM treatment can reduce the accumulation of succinate and protein expression of HIF-1α, α-SMA and FN, as well as inhibit cell migration. This is consistent with Wang et al.’s findings that the accumulation of succinic acid in lung fibroblasts stabilizes HIF-1α and promotes lung fibroblast activation^41^.

An extracellular signaling molecule, succinate, binds selectively to its receptor GPR91^21^. GPR91 is primarily expressed in kidney, followed by liver, spleen and small intestine^42^. However, the exact role of succinate-GPR91 axis in fibrosis development in patients with IPF remains unclear. The mRNA level and protein expression of GPR91 were significantly upregulated in fibrotic lung tissue from patients with IPF and BLM-induced pulmonary fibrosis in mice. Activation of GPR91 by succinate has been revealed to be critical for fibroblast activation and ECM production in murine intestinal fibrosis and non-alcoholic steatohepatitis (NASH) -associated fibrosis^31,43^. Based on immunohistochemistry, GPR91 expression was predominantly found in fibrotic lung lesions of IPF patients and lung fibrosis in mice induced by BLM. The expression of GPR91 was consistent with that fibrostic markers of α-SMA and FN. Moreover, GPR91-deficiency alleviates the lung fibrosis and alveolitis induced by BLM in mice. Based on these results, succinate-GPR91 signaling plays an important role in lung alveolitis, myofibroblast activation, and ECM deposition. Furthermore, succinate GPR91 signaling may be an important target for lung fibrosis intervention. In order for fibroblast differentiation and ECM deposition to occur, mitogen-activated protein kinases (MAPKs), extracellular signal-regulated kinases (ERKs), and p38 kinases play key roles^44–47^. Studies have demonstrated that succinate-GPR91 signaling activates ERK-1/2 in several cell lines^48,49^. We found succinate increased the ERK-1/2 phosphorylation and p38 phosphorylation MAPKs and promoted TGF-β induced α-SMA and FN expression of NHLFs. Both GPR91 antagonist NF-56-EJ40 and ERK inhibitor FR180204 inhibited succinate-induced ERK phosphorylation of NHLF and succinate-promoted lung fibroblast activation. In conclusion, succinate can promote the activation of lung fibroblasts by activating ERK pathway through GPR91.

The current study had several limitations. First, a selection bias may have been introduced due to the IPF cohort being drawn from a single outpatient center. Therefore, future studies are needed to analyze the relationship between serum succinate levels and treatment outcomes. Second, the cut-off succinate value for disease progression was exclusive to this study population. The optimal cutoff value for the general population needs to be validated further. Lastly, the detailed mechanism of the role of succinate in promoting pulmonary fibrosis and the role of succinate/GPR91 signaling pathway in regulating fibroblast activation still needs further study.

## Conclusion

Succinate, which acts as an extracellular signaling molecule in the regulation of pulmonary fibrosis, is a promising biomarker for diagnosis and prognosis of IPF. Activation of the succinate-GPR91 axis is associated with pulmonary fibrosis, and the accumulation of succinate may promote fibroblast activation through ERK pathway and pulmonary fibrosis. The succinate-GPR91 axis may be a potential therapeutic target for IPF and needs further study.

### Supplementary Information

Supplementary Figures.

Supplementary Tables.

Supplementary Information.

## Data Availability

The raw data supporting the conclusions of this article will be made available via the author’s email address (mengjie@csu.edu.cn), without undue reservation.

## Abbreviations

IPF: Idiopathic pulmonary fibrosis
Suc: Succinate
GPR91: G protein-coupled receptor 91
TCA cycle: Tricarboxylic acid cycle
DMM: Dimethyl malonate
ECM: Extracellular matrix
TGF-β: Transforming growth factor-β
BLM: Bleomycin
BALF: Bronchoalveolar lavage fluid
H&E: Hematoxylin and eosin
HR: Hazard ratio
OR: Odds ratio
CI: Confidence interval
AUC: Area under the curve
ROC: Receiver operating characteristic curve
BMI: Body mass index
PaO_2_: Arterial oxygen pressure
%FVC: Percent predicted FVC
%DLCO: Percent predicted diffusing capacity of the lung for carbon monoxide
GAP: Gender-age-physiology index
NHLFs: Normal human lung fibroblasts
HLF: Human lung fibroblasts derived from IPF
FN: Fibronectin
α-SMA: Alpha smooth muscle actin
HIF-1α: Hypoxia-inducing factor 1α
ERK1/2: Extracellular signal-regulated kinases
p-ERK: Phosphorylation extracellular signal-regulated kinases
p-38: P38 mitogen-activated protein kinase
p-p38: Phosphorylated p38 mitogen-activated protein kinase
