# Supplementary material for: Succinate promotes pulmonary fibrosis through GPR91 and predicts death in idiopathic pulmonary fibrosis
Source: Sci Rep. 2024 Jun 22;14:14376. doi: 10.1038/s41598-024-64844-5 (PMC11193722; doi:10.1038/s41598-024-64844-5)
Supplement: Supplementary file 1 — Supplementary Figures. [file 41598_2024_64844_MOESM1_ESM.doc]

A

B

C

D

E

Supplemental Figure 1 Correlations between succinate level and the clinical parameters, such as age (A) body mass index (BMI) (B), arterial partial pressure of oxygen (PaO_2_) (C), percent predicted forced vital capacity (%FVC) (D), and percent predicted diffusing capacity of the lung for carbon monoxide (%DLCO) (E) were analyzed by using the Pearson correlation coefficient.


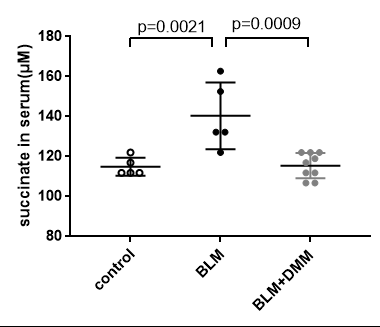

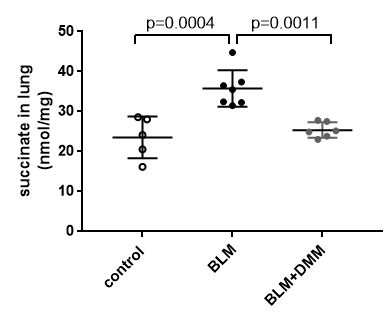


A

B

C

Supplemental Figure 2 Succinate level was significantly increased in fibrotic lung tissues from IPF patients(A). Succinate levels in serum (B) and lung tissues (C) were significantly down-regulated in BLM+DMM group compared to BLM model group.


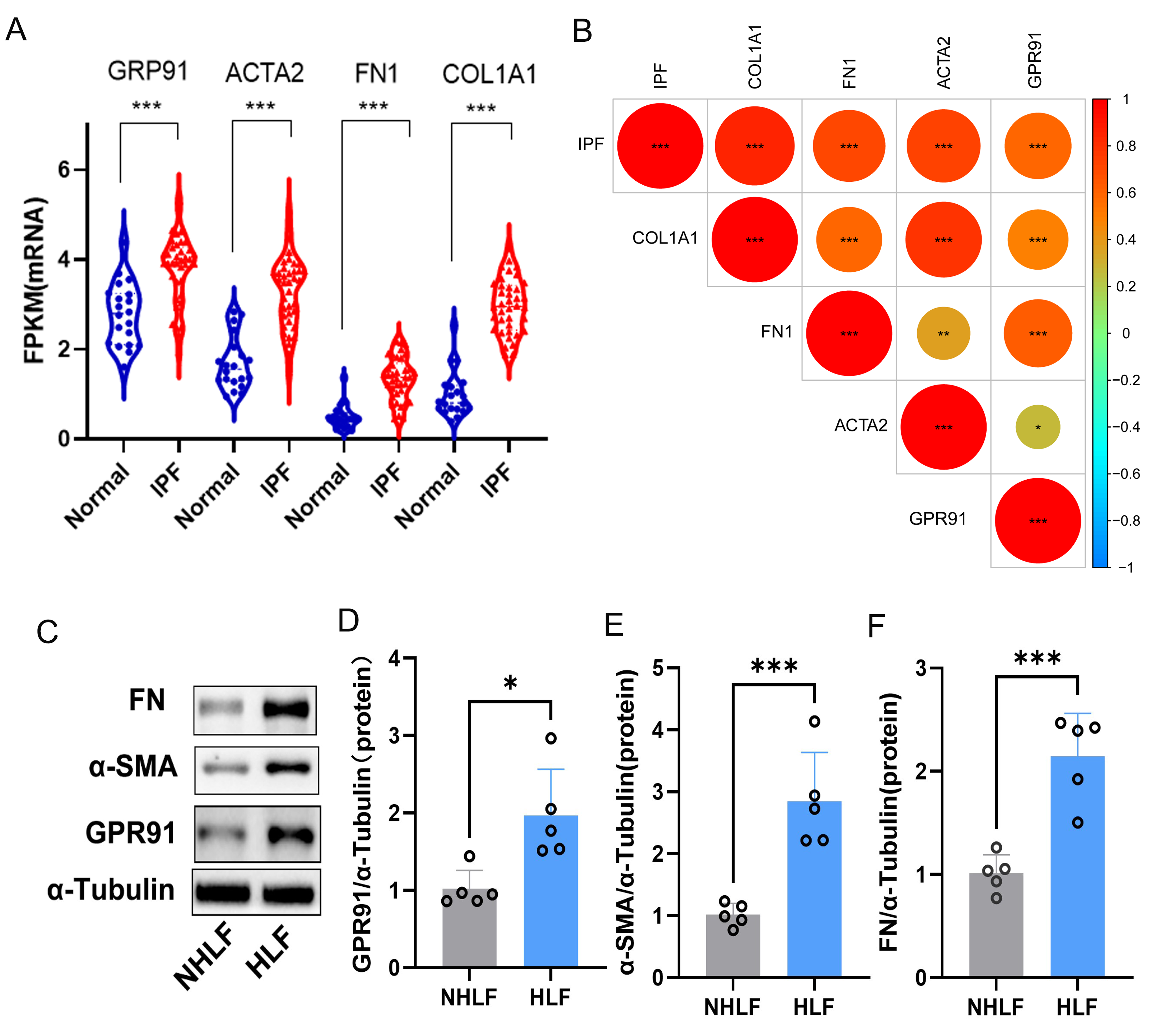


Supplemental Figure 3 Expression of GPR91 in IPF lung fibroblasts. The mRNA expression levels (A) of GPR91, ACTA2(α-SMA), FN1 (FN), COL1A1 and their co-expression correlation (B) (Pearson correlation) in the GSE17978 database. Western blot and analysis of the expression of GPR91, a-SMA and FN in IPF lung fibroblasts (HLF) and normal human lung fibroblasts (NHLF) (C-F).


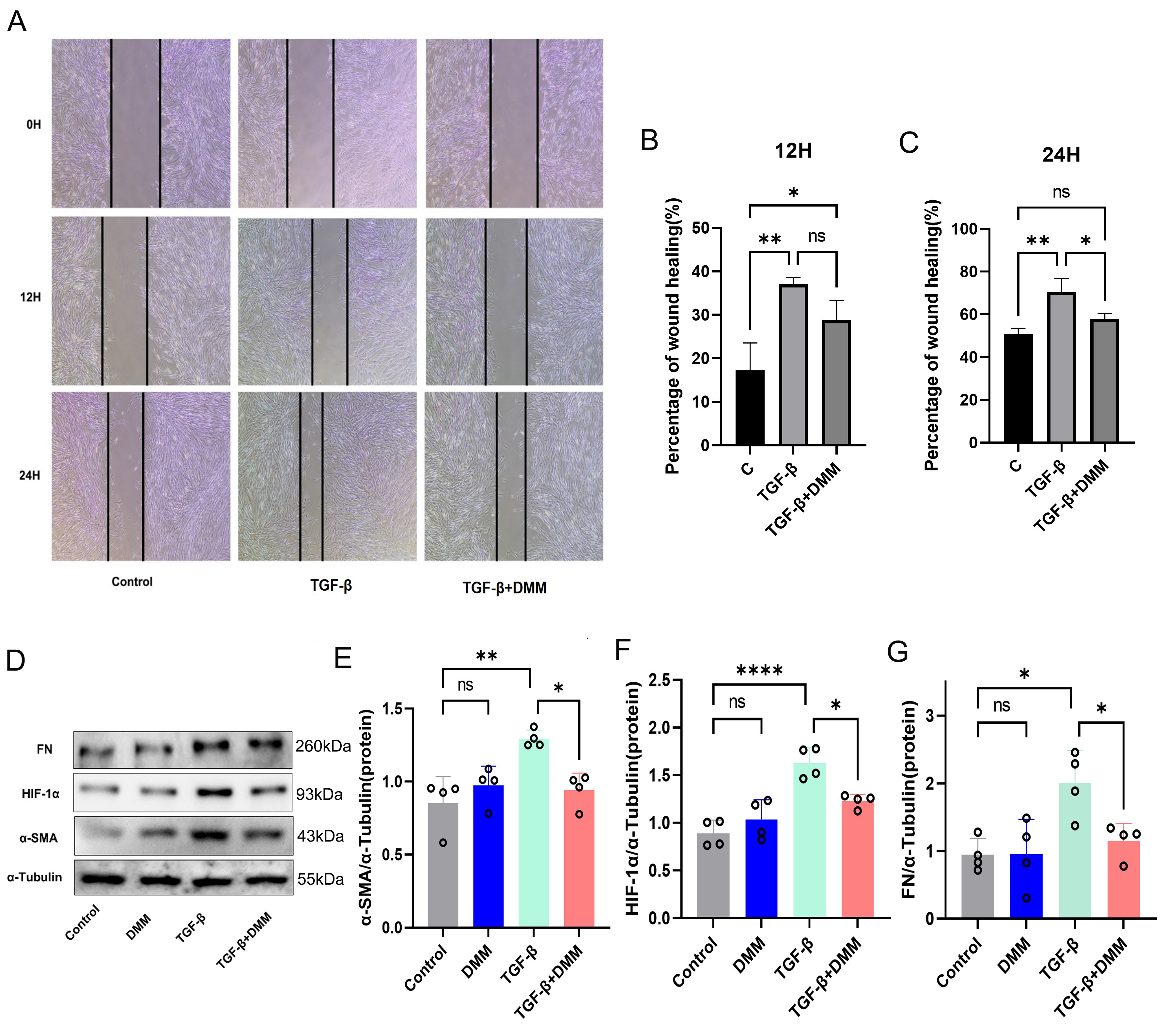


Supplemental Figure 4 DMM treatment inhibited TGFβ-induced migration and activation of normal human lung fibroblasts (NHLF). The effect of DMM treatment on TGF induced fibroblast migration (A) and cell fusion rate (B, C) were measured in different time by cell scratch assay. Western blotting (D) and quantitative analysis of α-SMA(E), HIF-1α(F), and FN(G) in normal human lung fibroblasts (NHLFs) treated pretreated with/without DMM for 2h and treated TGF-β for 24h.


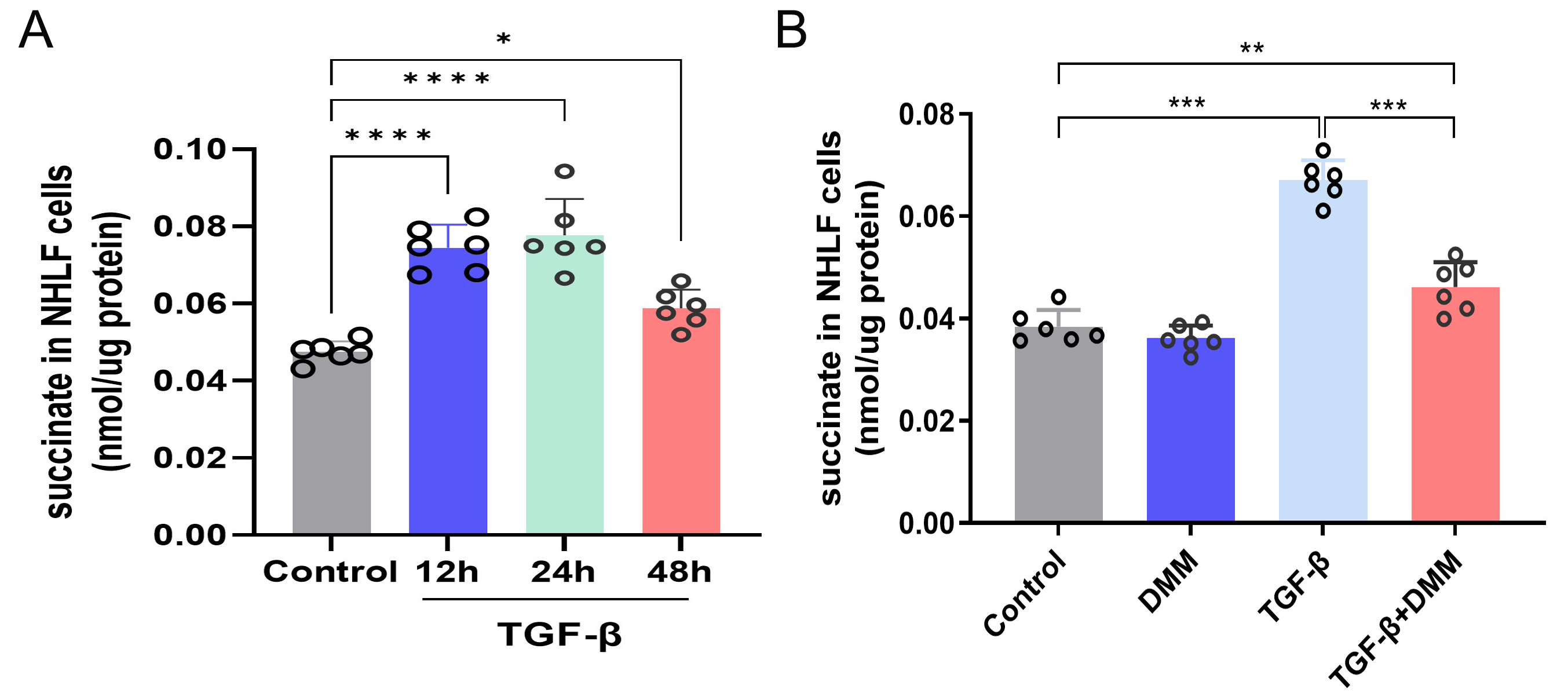


Supplemental Figure 5 DMM inhibits TGF-induced succiniate accumulation in normal human lung fibroblasts (NHLFs). TGF stimulates the level of succinate accumulation in NHLFs at different time points (A). Succinate levels in NHLFs treated pretreated with/without DMM for 2h and treated TGF-β for 24h (B).
