# Supplementary material for: Succinate promotes pulmonary fibrosis through GPR91 and predicts death in idiopathic pulmonary fibrosis
Source: Sci Rep. 2024 Jun 22;14:14376. doi: 10.1038/s41598-024-64844-5 (PMC11193722; doi:10.1038/s41598-024-64844-5)
Supplement: Supplementary file 2 — Supplementary Tables. [file 41598_2024_64844_MOESM2_ESM.docx]

Supplemental Table 1.Baseline demographic and clinical charateristics of patients with healthy control and IPF for metabolic profiling

| **Characteristic** | **Healthy control(n=6)** | **IPF patients(n=10)** | **p-value** |
| --- | --- | --- | --- |
| Age,yr,mean(SD) | 59.17(9.99) | 63.20(12.13) | 0.5059 |
| Gender,male(%) | 4(66.67） | 7(70.00) | 0.8981 |
| PaO2,mmHg,mean(SD) | - | 72.10(9.71) | - |
| %FVC, %, mean(SD) | - | 75.33(13.79) | - |
| %DLCO, %, mean(SD) | - | 53.12(10.56) | - |
| GAP stage(I/II/III) | - | 6/3/1 | - |

Supplemental Table 2. Sample size calculation method

| Formula | α | 1-β | Standard deviation ($\sigma$*)* | Difference in means ($\epsilon$) | $\kappa$(n1=$\kappa$n2) | $n1$  (IPF) | $n2$  (Control) |
| --- | --- | --- | --- | --- | --- | --- | --- |
| $n2=\frac{\left（ Z_{\alpha/2}+Z_{\beta} \right）^{2}\sigma^{2}(1+1/\kappa)}{\epsilon^{2}}$ | 0.05 | 0.8 | 10 | 12 | 3 | 51 | 17 |

Supplemental Table 3. Metabolic substance levels detected in serum

| Compounds | C13 | C14 | C15 | C16 | C46 | C22 | 51 | 73 | 49 | 62 | 94 | 16 | 50 | 77 | 55 | 1 |
| --- | --- | --- | --- | --- | --- | --- | --- | --- | --- | --- | --- | --- | --- | --- | --- | --- |
| L-Arginine | 1.74E+04 | 2.14E+04 | 1.90E+04 | 1.89E+04 | 1.65E+04 | 1.71E+04 | 1.28E+04 | 1.52E+04 | 1.91E+04 | 1.34E+04 | 1.51E+04 | 1.87E+04 | 2.87E+04 | 2.03E+04 | 2.19E+04 | 1.87E+04 |
| L-Cystine | 4.55E+03 | 5.00E+03 | 5.99E+03 | 4.95E+03 | 3.83E+03 | 3.83E+03 | 2.29E+03 | 3.14E+03 | 2.80E+03 | 1.60E+03 | 2.74E+03 | 1.68E+03 | 1.80E+03 | 3.40E+03 | 3.02E+03 | 1.95E+03 |
| Beta-Leucine | 1.09E+04 | 1.47E+04 | 1.23E+04 | 1.20E+04 | 9.18E+03 | 1.38E+04 | 9.97E+03 | 9.06E+03 | 1.04E+04 | 8.76E+03 | 8.92E+03 | 1.28E+04 | 1.47E+04 | 9.56E+03 | 1.08E+04 | 1.33E+04 |
| Lysine | 1.43E+04 | 1.51E+04 | 1.42E+04 | 1.77E+04 | 1.17E+04 | 1.70E+04 | 1.24E+04 | 1.18E+04 | 8.48E+03 | 7.46E+03 | 8.98E+03 | 7.81E+03 | 9.57E+03 | 8.45E+03 | 8.89E+03 | 1.09E+04 |
| Ornithine | 2.93E+04 | 2.93E+04 | 3.17E+04 | 3.30E+04 | 2.48E+04 | 2.85E+04 | 3.93E+04 | 2.87E+04 | 2.60E+04 | 2.51E+04 | 2.95E+04 | 2.56E+04 | 3.56E+04 | 3.08E+04 | 4.10E+04 | 3.42E+04 |
| D-Glutamine | 1.14E+04 | 1.23E+04 | 1.06E+04 | 1.13E+04 | 9.54E+03 | 1.23E+04 | 1.06E+04 | 1.12E+04 | 9.23E+03 | 7.83E+03 | 8.58E+03 | 9.31E+03 | 9.21E+03 | 1.05E+04 | 1.06E+04 | 2.72E+03 |
| L-Asparagine | 6.67E+03 | 1.10E+04 | 8.99E+03 | 1.08E+04 | 9.05E+03 | 1.16E+04 | 4.03E+03 | 5.81E+03 | 3.36E+03 | 3.02E+03 | 3.54E+03 | 3.28E+03 | 1.26E+04 | 1.00E+04 | 1.38E+04 | 1.24E+04 |
| Succinic acid | 1.89E+03 | 3.30E+03 | 2.50E+03 | 2.95E+03 | 2.26E+03 | 2.15E+03 | 4.84E+03 | 2.59E+03 | 2.60E+03 | 3.25E+03 | 4.89E+03 | 2.50E+03 | 3.04E+03 | 4.12E+03 | 3.78E+03 | 3.15E+03 |
| L-Serine | 1.14E+04 | 1.71E+04 | 1.46E+04 | 1.61E+04 | 1.54E+04 | 1.54E+04 | 9.38E+03 | 1.08E+04 | 7.20E+03 | 6.65E+03 | 7.66E+03 | 6.69E+03 | 2.31E+04 | 1.54E+04 | 1.76E+04 | 2.21E+04 |
| L-Threonine | 1.24E+04 | 2.36E+04 | 1.52E+04 | 1.84E+04 | 1.36E+04 | 1.80E+04 | 8.07E+03 | 8.90E+03 | 7.40E+03 | 5.77E+03 | 7.94E+03 | 9.43E+03 | 1.27E+04 | 1.49E+04 | 1.56E+04 | 1.16E+04 |
| L-Alanine | 1.42E+04 | 3.04E+04 | 2.11E+04 | 2.26E+04 | 1.67E+04 | 2.48E+04 | 2.90E+04 | 1.83E+04 | 2.12E+04 | 1.63E+04 | 1.50E+04 | 2.54E+04 | 2.35E+04 | 1.75E+04 | 2.55E+04 | 1.93E+04 |
| Adenine | 2.82E+00 | 5.23E+00 | 3.55E+00 | 2.58E+00 | 2.62E+00 | 2.17E+00 | 2.45E+00 | 2.47E+00 | 3.66E-01 | 1.58E+00 | 3.32E+00 | 2.28E+00 | 3.72E+00 | 4.63E+00 | 7.70E+00 | 4.25E+00 |
| Inosine | 8.84E+01 | 9.84E+01 | 4.30E+02 | 6.19E+01 | 3.50E+02 | 5.78E+01 | 1.07E+02 | 7.83E+00 | 2.12E+00 | 4.51E+00 | 1.03E+01 | 8.24E+00 | 2.14E+03 | 1.74E+02 | 5.35E+01 | 1.27E+01 |
| Phenyllactate | 1.80E+01 | 3.18E+01 | 1.31E+01 | 1.59E+01 | 9.82E+00 | 1.34E+01 | 4.12E+01 | 3.09E+00 | 2.55E+01 | 7.77E+00 | 3.29E+01 | 6.01E+01 | 2.78E+01 | 2.78E+01 | 6.90E+01 | 5.13E+01 |
| Citric acid | 2.12E+03 | 2.11E+03 | 1.88E+03 | 1.68E+03 | 2.32E+03 | 1.47E+03 | 4.30E+03 | 2.68E+03 | 2.31E+03 | 3.25E+03 | 7.13E+03 | 2.57E+03 | 2.32E+03 | 4.22E+03 | 5.37E+03 | 1.83E+03 |
| L-Lactate | NA | NA | NA | NA | NA | NA | NA | NA | NA | NA | NA | 1.08E+03 | 1.14E+03 | 6.45E+02 | 3.05E+02 | 1.70E+03 |
| ADP | NA | NA | NA | NA | NA | NA | 6.34E+02 | 2.86E+02 | 2.34E+02 | 3.33E+02 | 2.10E+02 | 3.76E+02 | NA | NA | NA | NA |
| Continued |  |  |  |  |  |  |  |  |  |  |  |  |  |  |  |  |
|  |  |  |  |  |  |  |  |  |  |  |  |  |  |  |  |  |
| Continued |  |  |  |  |  |  |  |  |  |  |  |  |  |  |  |  |
| Compounds | C13 | C14 | C15 | C16 | C46 | C22 | 51 | 73 | 49 | 62 | 94 | 16 | 50 | 77 | 55 | 1 |
| Uracil | 9.12E+01 | 9.37E+01 | 1.10E+02 | 7.30E+01 | 8.33E+01 | 6.77E+01 | 1.13E+02 | 1.30E+02 | 9.85E+01 | 1.10E+02 | 3.55E+02 | 8.84E+01 | 9.46E+01 | 1.30E+02 | 1.35E+02 | 7.30E+01 |
| Guanosine | 1.32E+01 | 1.19E+01 | 4.45E+01 | 8.20E+00 | 1.91E+01 | 1.06E+01 | 1.07E+01 | 2.13E-01 | 1.07E+01 | 1.07E+01 | 1.07E+01 | 1.07E+01 | 1.55E+01 | 2.31E+01 | 3.23E+00 | 7.65E-01 |
| Alpha-Ketoglutaric acid | 3.84E+03 | 3.17E+03 | 7.32E+03 | 3.36E+03 | 2.28E+03 | 3.01E+03 | 4.77E+03 | 4.26E+03 | 2.88E+03 | 4.31E+03 | 1.05E+04 | 5.36E+03 | 3.15E+03 | 2.06E+03 | 3.86E+03 | 1.45E+04 |
| L-Glutamate | 3.68E+04 | 5.95E+04 | 5.89E+04 | 3.76E+04 | 2.81E+04 | 4.37E+04 | 3.93E+04 | 4.01E+04 | 4.12E+04 | 3.57E+04 | 4.37E+04 | 4.94E+04 | 8.86E+04 | 3.79E+04 | 7.12E+04 | 1.50E+05 |
| L-Tyrosine | 1.24E+04 | 2.45E+04 | 2.02E+04 | 1.89E+04 | 1.35E+04 | 1.76E+04 | 2.56E+04 | 1.57E+04 | 1.43E+04 | 1.37E+04 | 2.13E+04 | 2.80E+04 | 1.91E+04 | 2.04E+04 | 1.27E+04 | 2.29E+04 |
| D-Glucose 6-phosphate | 6.55E+00 | 7.47E+00 | 3.67E+00 | 5.23E+00 | 7.48E+00 | 8.58E+00 | 2.11E+01 | 3.23E+00 | 8.61E+00 | 5.79E+00 | 4.72E+00 | 5.95E+00 | 1.37E+00 | 3.75E+00 | 1.81E+00 | 1.15E+00 |
| Fructose 1,6-bisphosphate | 4.25E+01 | 4.04E+01 | 3.51E+01 | NA | NA | NA | NA | NA | NA | NA | NA | NA | NA | NA | NA | NA |
| Glycerol 3-phosphate | 2.78E+02 | 3.40E+02 | 1.00E+02 | 2.72E+02 | 1.99E+02 | 3.19E+02 | 1.02E+02 | 8.90E+01 | 8.95E+01 | 5.17E+01 | 5.88E+01 | 5.38E+01 | 1.03E+02 | 2.19E+02 | 7.61E+01 | 6.33E+01 |
| Phosphoenolpyruvic acid | NA | NA | NA | NA | NA | 2.14E+01 | 5.53E+01 | 4.61E+01 | 4.47E+01 | 3.34E+01 | 3.02E+01 | 2.75E+01 | NA | NA | NA | NA |
| Pyruvic acid | 2.18E+02 | 2.32E+02 | 2.58E+02 | 3.29E+02 | 2.39E+02 | 2.18E+02 | 8.46E+02 | 7.56E+02 | 3.97E+02 | 9.92E+02 | 6.56E+02 | 9.94E+02 | 2.44E+02 | 2.61E+02 | 3.07E+02 | 3.46E+02 |
| D-Ribulose 5-phosphate | NA | NA | NA | NA | NA | NA | 9.56E+01 | 1.08E+02 | 9.78E+01 | 9.55E+01 | 9.31E+01 | 9.37E+01 | NA | NA | NA | NA |
| Dihydroxyacetone phosphate | NA | NA | NA | NA | NA | NA | 4.07E+02 | 2.98E+02 | 2.64E+02 | 2.01E+02 | 1.64E+02 | 1.87E+02 | NA | NA | NA | NA |
| Isocitric acid | 3.48E+02 | 2.34E+02 | 2.09E+02 | 1.45E+02 | 1.37E+02 | 1.32E+02 | 8.18E+02 | 3.65E+02 | 4.12E+02 | 4.01E+02 | 8.86E+02 | 2.85E+02 | 2.14E+02 | 3.73E+02 | 3.60E+02 | 1.36E+02 |
| AMP | NA | NA | NA | NA | NA | NA | 1.22E+03 | 1.38E+02 | 4.67E+02 | 1.49E+02 | 8.13E+01 | 1.71E+02 | NA | NA | NA | NA |
| IMP | NA | NA | NA | NA | NA | NA | 2.10E+02 | 1.26E+02 | 8.03E+01 | 4.52E+01 | NA | 2.28E+01 | NA | NA | NA | NA |
| UDP-GlcNAc | 1.07E+01 | 1.27E+01 | 2.06E+01 | 2.48E+01 | 1.16E+01 | NA | 2.67E+01 | 4.86E+01 | 1.42E+01 | 4.59E+01 | 2.43E+01 | 4.00E+01 | NA | NA | 1.19E+01 | NA |
| BPG | 1.09E+02 | 1.02E+02 | 9.56E+01 | 9.32E+01 | 9.04E+01 | 8.79E+01 | 1.24E+02 | 9.40E+01 | 9.27E+01 | 1.07E+02 | 2.53E+02 | 4.04E+02 | 8.45E+01 | 8.23E+01 | 8.27E+01 | 8.19E+01 |
| Continued |  |  |  |  |  |  |  |  |  |  |  |  |  |  |  |  |
|  |  |  |  |  |  |  |  |  |  |  |  |  |  |  |  |  |
| Continued |  |  |  |  |  |  |  |  |  |  |  |  |  |  |  |  |
| Compounds | C13 | C14 | C15 | C16 | C46 | C22 | 51 | 73 | 49 | 62 | 94 | 16 | 50 | 77 | 55 | 1 |
| ATP | NA | NA | NA | NA | NA | NA | 2.54E+02 | 3.89E+02 | 1.30E+02 | 3.47E+02 | 4.90E+02 | 5.49E+02 | NA | NA | NA | NA |
| 2-Phospho-D-glyceric acid | NA | NA | NA | NA | NA | NA | 8.55E+02 | 2.95E+02 | 6.54E+02 | 3.31E+02 | 2.54E+02 | 3.56E+02 | NA | NA | NA | NA |
| Guanosine diphosphate | NA | NA | NA | NA | NA | NA | 1.63E+02 | 6.13E+01 | 3.70E+01 | 5.54E+01 | 4.85E+01 | 6.69E+01 | NA | NA | NA | NA |
| Argininosuccinic acid | 2.51E+02 | 2.45E+02 | 2.52E+02 | 2.71E+02 | 2.45E+02 | 2.47E+02 | 2.54E+02 | 2.44E+02 | 2.54E+02 | 2.47E+02 | 2.85E+02 | 2.63E+02 | 2.58E+02 | 2.81E+02 | 2.83E+02 | 2.52E+02 |
| Phosphorylethanolamine | 2.90E+02 | 3.13E+02 | 1.72E+02 | 2.63E+02 | 4.72E+02 | 1.95E+02 | 1.06E+03 | 5.16E+02 | 4.74E+02 | 6.55E+02 | 4.46E+02 | 5.21E+02 | 6.12E+02 | 6.12E+02 | 6.12E+02 | 6.12E+02 |
| L-Citrulline | 6.07E+03 | 9.82E+03 | 9.96E+03 | 7.70E+03 | 7.37E+03 | 7.65E+03 | 3.00E+03 | 3.20E+03 | 3.48E+03 | 1.99E+03 | 3.68E+03 | 3.15E+03 | 7.83E+03 | 8.17E+03 | 1.14E+04 | 2.97E+03 |
| Fumaric acid | 5.51E+02 | 6.70E+02 | 6.22E+02 | 6.54E+02 | 6.46E+02 | 5.42E+02 | 7.34E+02 | 4.98E+02 | 4.74E+02 | 5.55E+02 | 8.09E+02 | 5.45E+02 | 5.24E+02 | 5.62E+02 | 7.10E+02 | 5.13E+02 |

Supplemental Table 4. Differences in metabolic substance levels

| Compounds | Pvalue | Fold_Change | Log2FC | Type |
| --- | --- | --- | --- | --- |
| L-Arginine | 0.993379119 | 0.9992 | -0.0012 | insig |
| L-Cystine | 0.000302187 | 0.5208 | -0.9412 | down |
| Beta-Leucine | 0.230008623 | 0.8906 | -0.1672 | insig |
| Lysine | 0.000512355 | 0.6312 | -0.6638 | down |
| Ornithine | 0.336704098 | 1.0727 | 0.1012 | insig |
| D-Glutamine | 0.025434997 | 0.8012 | -0.3198 | down |
| L-Asparagine | 0.140957904 | 0.7411 | -0.4323 | insig |
| Succinic acid | 0.016550278 | 1.3861 | 0.471 | up |
| L-Serine | 0.30140607 | 0.843 | -0.2464 | insig |
| L-Threonine | 0.007995599 | 0.6068 | -0.7207 | down |
| Continued |  |  |  |  |
| Compounds | Pvalue | Fold_Change | Log2FC | Type |
| L-Alanine | 0.843940001 | 0.974 | -0.038 | insig |
| Adenine | 0.886864056 | 1.0357 | 0.0506 | insig |
| Inosine | 0.754083284 | 1.3923 | 0.4775 | insig |
| Phenyllactate | 0.034301373 | 2.0372 | 1.0266 | up |
| Citric acid | 0.011570438 | 1.865 | 0.8992 | up |
| L-Lactate | - | - | - | insig |
| ADP | - | - | - | insig |
| Uracil | 0.107672054 | 1.5352 | 0.6184 | insig |
| Guanosine | 0.225170942 | 0.4783 | -1.064 | down |
| Alpha-Ketoglutaric acid | 0.245691417 | 1.4543 | 0.5403 | up |
| L-Glutamate | 0.235189665 | 1.3549 | 0.4382 | insig |
| L-Tyrosine | 0.542096891 | 1.0865 | 0.1197 | insig |
| D-Glucose 6-phosphate | 0.715746696 | 0.8852 | -0.1759 | insig |
| Fructose 1,6-bisphosphate | - | - | - | insig |
| Glycerol 3-phosphate | 0.004781014 | 0.3605 | -1.4719 | down |
| Phosphoenolpyruvic acid | - | 1.8486 | 0.8864 | insig |
| Pyruvic acid | 0.00721999 | 2.3271 | 1.2185 | up |
| D-Ribulose 5-phosphate | - | - | - | insig |
| Dihydroxyacetone phosphate | - | - | - | insig |
| Isocitric acid | 0.020013276 | 2.1146 | 1.0804 | up |
| AMP | - | - | - | insig |
| IMP | - | - | - | insig |
| UDP-GlcNAc | 0.051920162 | 1.8788 | 0.9098 | insig |
| BPG | 0.220033219 | 1.4607 | 0.5467 | insig |
| ATP | - | - | - | insig |
| 2-Phospho-D-glyceric acid | - | - | - | insig |
| Guanosine diphosphate | - | - | - | insig |
| Argininosuccinic acid | 0.123531728 | 1.0405 | 0.0573 | insig |
| Phosphorylethanolamine | 0.016031734 | 2.1538 | 1.1069 | up |
| L-Citrulline | 0.015663943 | 0.6041 | -0.7271 | insig |
| Fumaric acid | 0.619598742 | 0.9647 | -0.0518 | insig |

Supplemental Table 5. Logistic Regression Analysis of serum succinate for Disease Progression

| **Characteristic** | **Univariate analysis** | | | | **Multivariate analysis** | | |
| --- | --- | --- | --- | --- | --- | --- | --- |
|  | OR | 95% CI | p value | | OR | 95% CI | p value |
| Model^a^ |  |  |  |  | |  |  |
| Age | 1.055 | 0.990-1.124 | 0.098 |  | |  |  |
| Gender, male | 1.397 | 0.425-4.593 | 0.582 |  | |  |  |
| BMI | 0.788 | 0.645-0.962 | 0.019 | 0.845 | | 0.673-1.061 | 0.147 |
| Smoke | 1.636 | 0.559-4.790 | 0.369 |  | |  |  |
| PaO_2_ | 0.949 | 0.895-1.007 | 0.082 |  | |  |  |
| % FVC | 0.945 | 0.899-0.994 | 0.027 | 0.968 | | 0.907-1.034 | 0.334 |
| % DLCO | 0.915 | 0.855-0.980 | 0.011 | 0.958 | | 0.880-1.043 | 0.322 |
| Succinate | 1.033 | 1.009-1.056 | 0.006 | 1.030 | | 1.006-1.055 | 0.015 |
| Model^b^ |  |  |  |  | |  |  |
| BMI＜24kg/m^2^ | 4.442 | 1.412-13.973 | 0.011 | 4.410 | | 1.035-18.794 | 0.045 |
| GAP stage | 3.689 | 1.419-9.596 | 0.007 | 4.630 | | 1.402-15.287 | 0.012 |
| Succinate^high^ | 8.531 | 2.533-28.729 | 0.001 | 13.087 | | 2.819-60.761 | 0.001 |

^a^ BMI and succinate as continuous variables.

^b^ BMI and succinate as categorical variables.

Abbreviations: OR, odds ratio; CI, confidence interval; BMI, body mass index; PaO_2_, arterial oxygen pressure; %FVC, percent predicted FVC; %DLCO, percent predicted diffusing capacity of the lung for carbon monoxide; GAP, gender-age-physiology index; Succinate^high^, serum succinate ≥ 117.5μM.

Supplemental Table 6. Cox Proportional Hazards Regression Analysis of serum succinate for all-cause Mortality

| **Characteristic** | **Univariate analysis** | | | **Multivariate analysis** | | |
| --- | --- | --- | --- | --- | --- | --- |
|  | HR | 95% CI | p value | HR | 95% CI | p value |
| Model^a^ |  |  |  |  |  |  |
| Age | 1.034 | 0.983-1.087 | 0.195 |  |  |  |
| Gender, male | 1.009 | 0.395-2.579 | 0.985 |  |  |  |
| BMI | 0.850 | 0.735-0.984 | 0.030 | 0.935 | 0.805-1.085 | 0.375 |
| Smoke | 0.954 | 0.412-2.210 | 0.913 |  |  |  |
| PaO_2_ | 0.981 | 0.943-1.020 | 0.331 |  |  |  |
| % FVC | 0.957 | 0.927-0.987 | 0.006 | 0.972 | 0.931-1.016 | 0.209 |
| % DLCO | 0.924 | 0.883-0.966 | 0.001 | 0.954 | 0.896-1.016 | 0.145 |
| Succinate | 1.018 | 1.005-1.030 | 0.005 | 1.017 | 1.003-1.030 | 0.016 |
| Model^b^ |  |  |  |  |  |  |
| BMI＜24kg/m^2^ | 2.687 | 1.121-6.439 | 0.027 | 2.281 | 0.935-5.567 | 0.070 |
| GAP stage | 2.378 | 1.362-4.153 | 0.002 | 2.437 | 1.359-4.370 | 0.003 |
| Succinate^high^ | 3.288 | 1.280-8.448 | 0.013 | 3.418 | 1.308-8.927 | 0.012 |

^a^ BMI and succinate as continuous variables.

^b^ BMI and succinate as categorical variables.

Abbreviations: HR, hazard ratio; CI, confidence interval; BMI, body mass index; PaO_2_, arterial oxygen pressure; % FVC, percent predicted FVC; % DLCO, percent predicted diffusing capacity of the lung for carbon monoxide; GAP, gender-age-physiology index; Succinate^high^, serum succinate ≥ 117.5μM.
