# Supplementary material for: Succinate promotes pulmonary fibrosis through GPR91 and predicts death in idiopathic pulmonary fibrosis
Source: Sci Rep. 2024 Jun 22;14:14376. doi: 10.1038/s41598-024-64844-5 (PMC11193722; doi:10.1038/s41598-024-64844-5)

Figure 4A

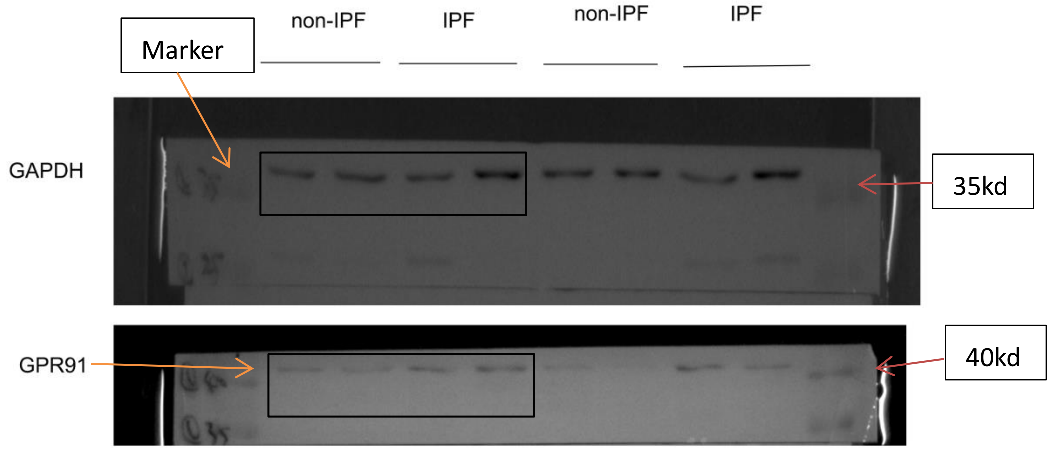

Figure 4A

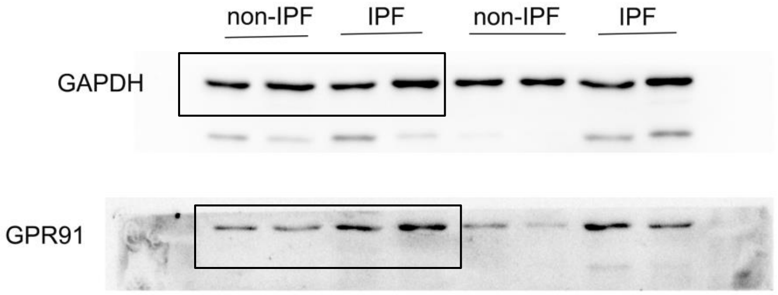

Figure 5C

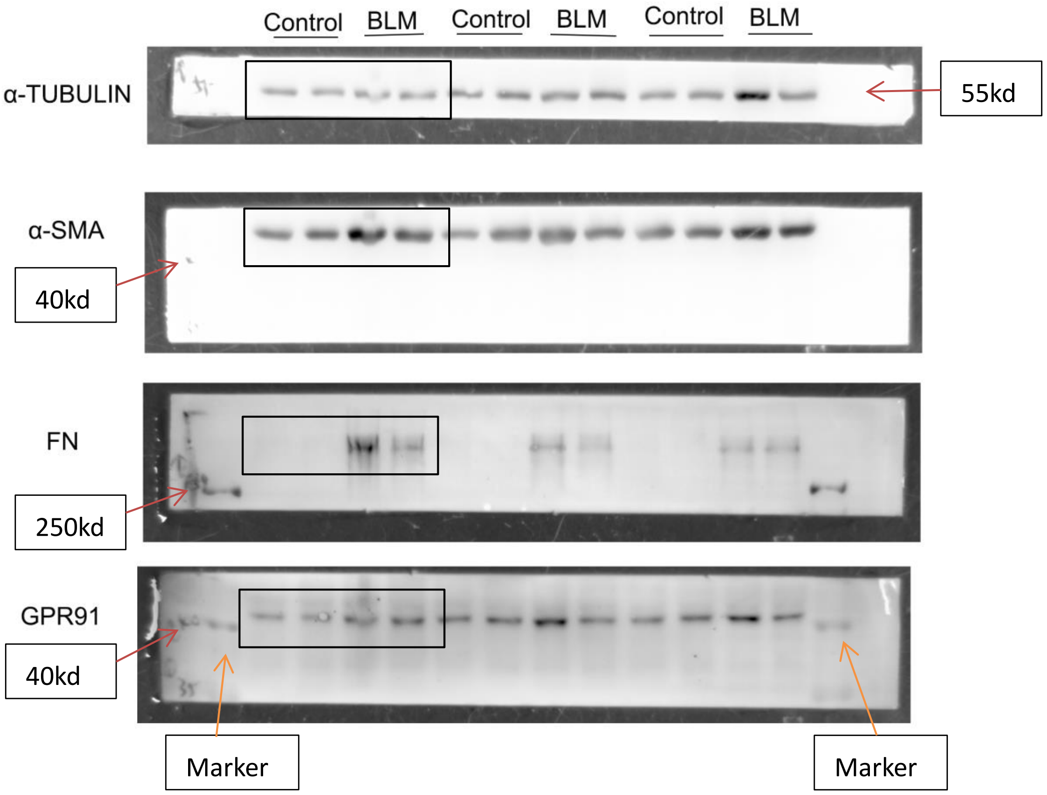

Figure 5C

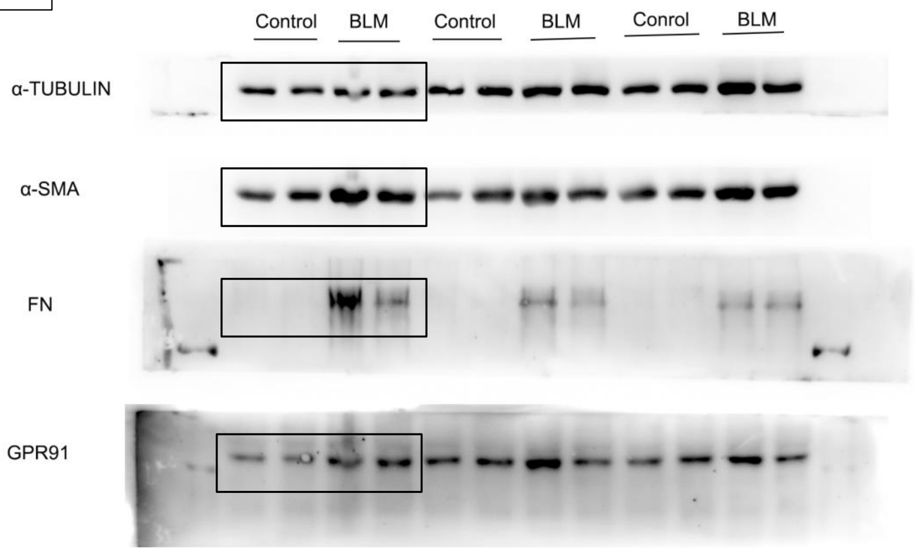

Figure 6G

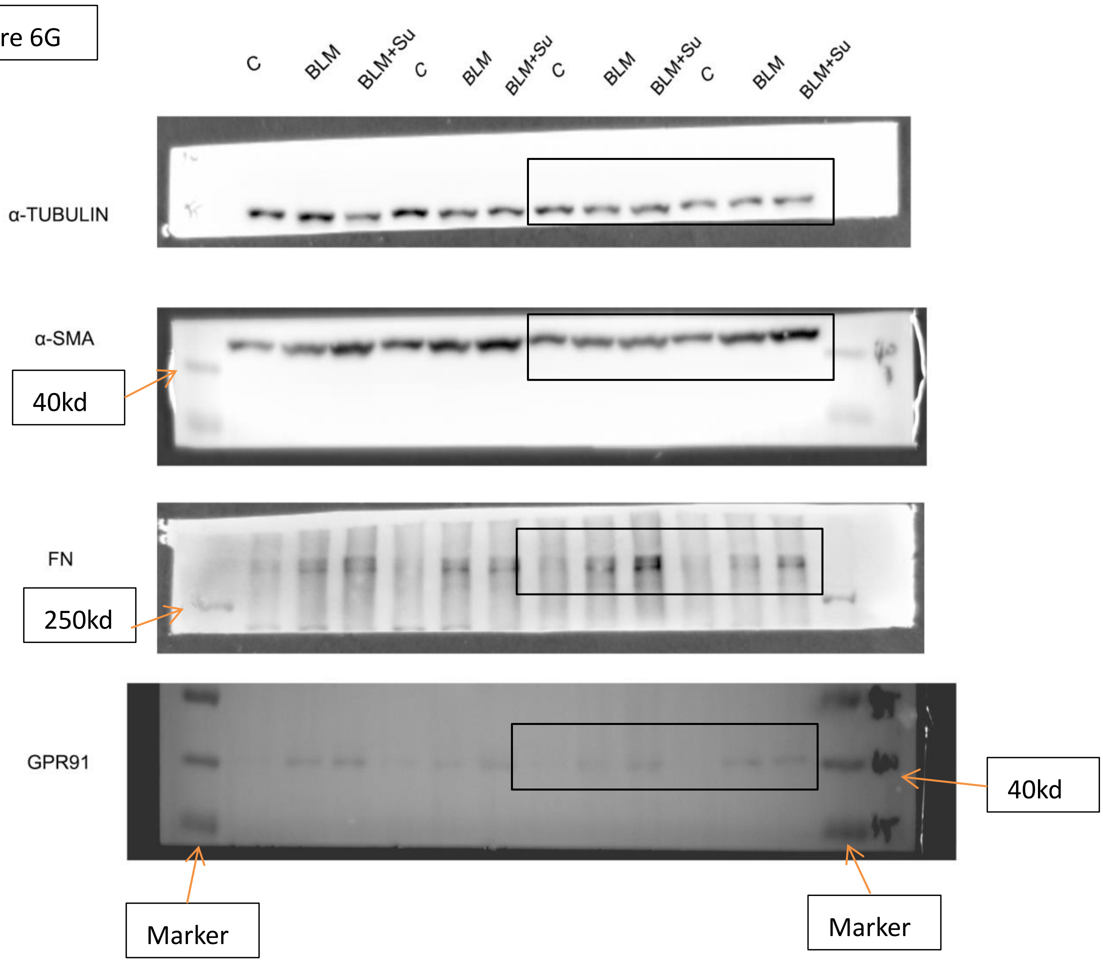

Figure 6G

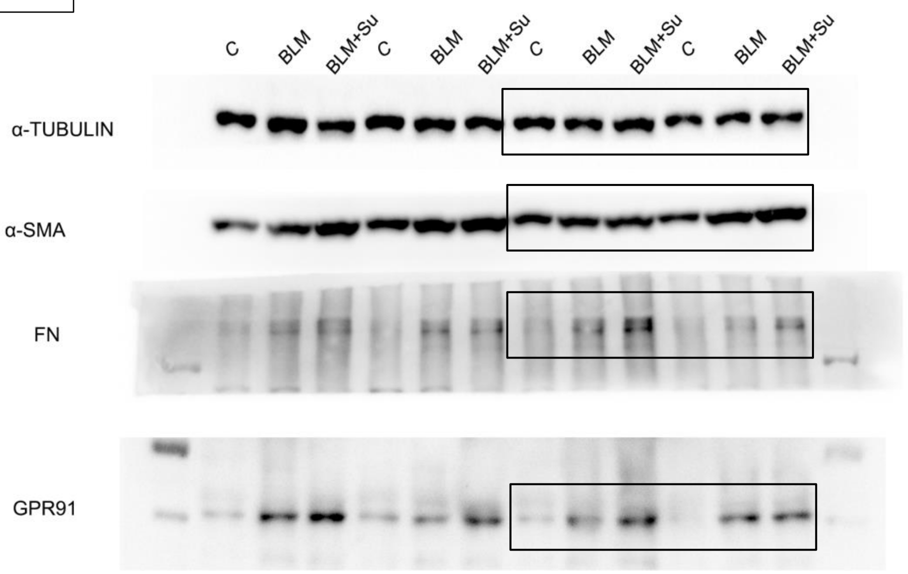

Figure 7G

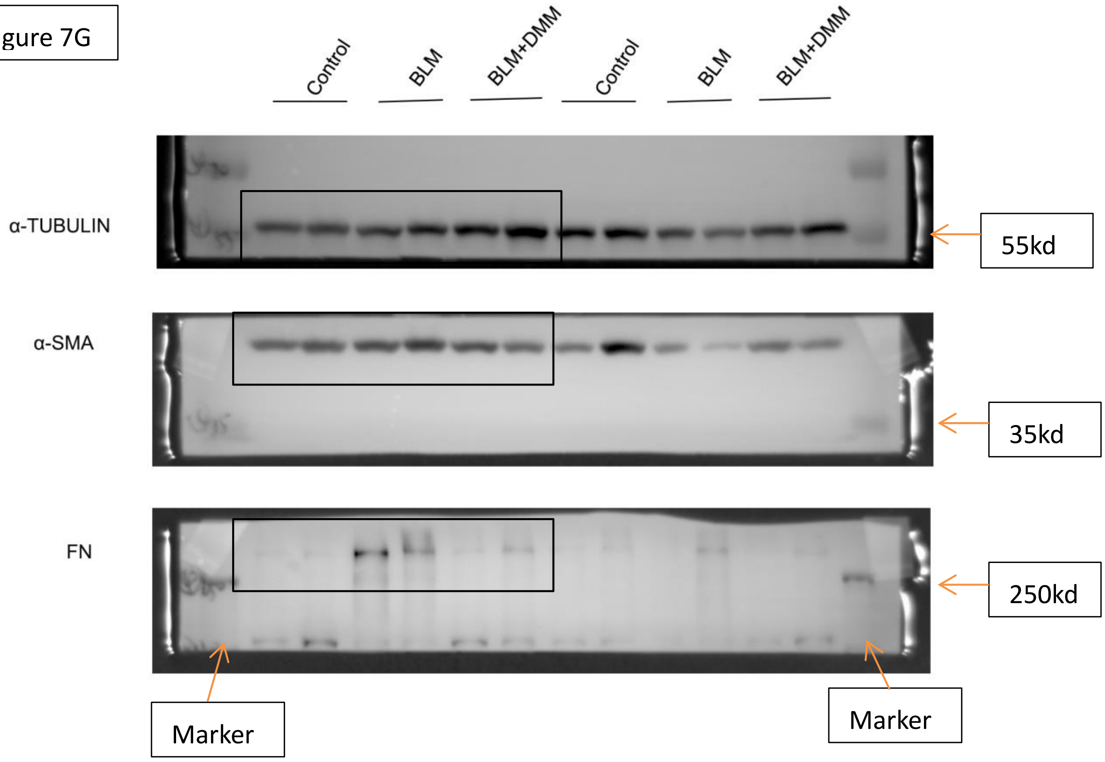

Figure 7G

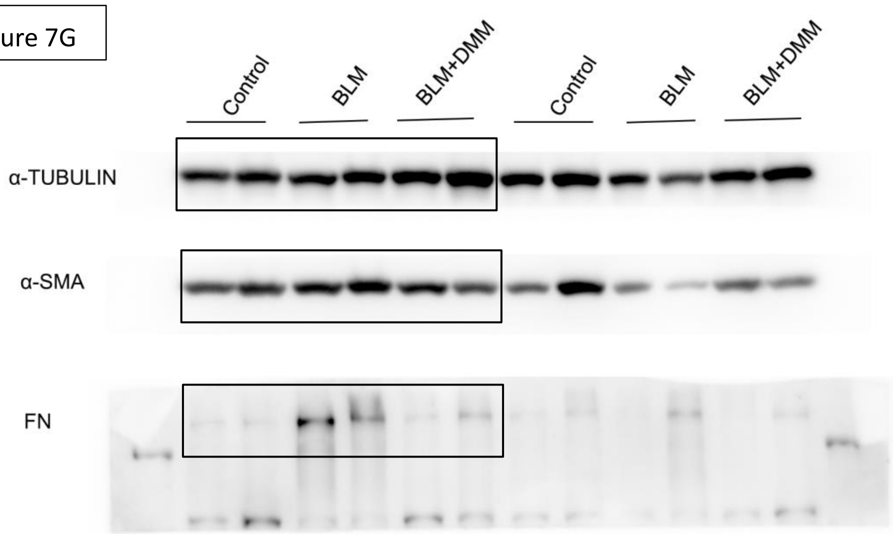

Figure 8G

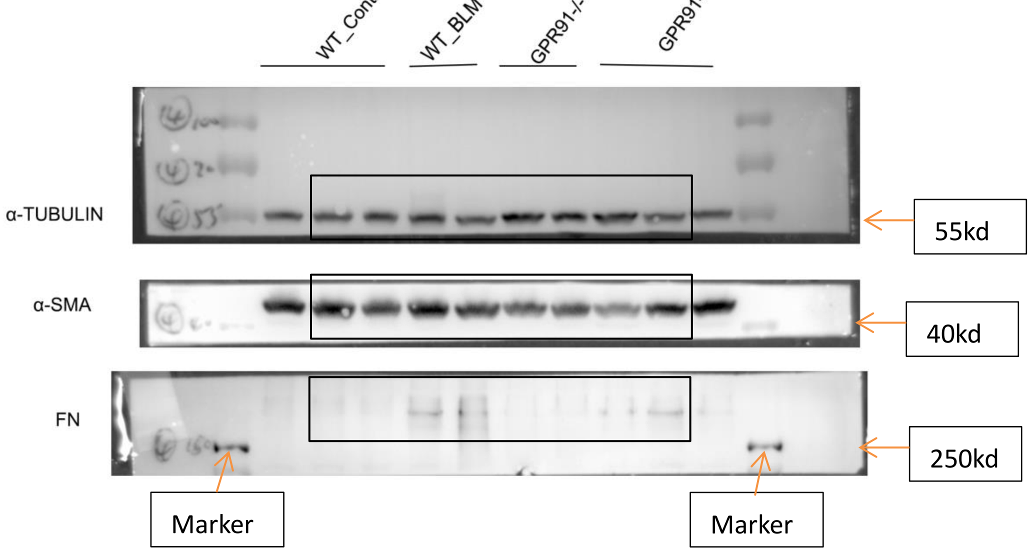

Figure 8G

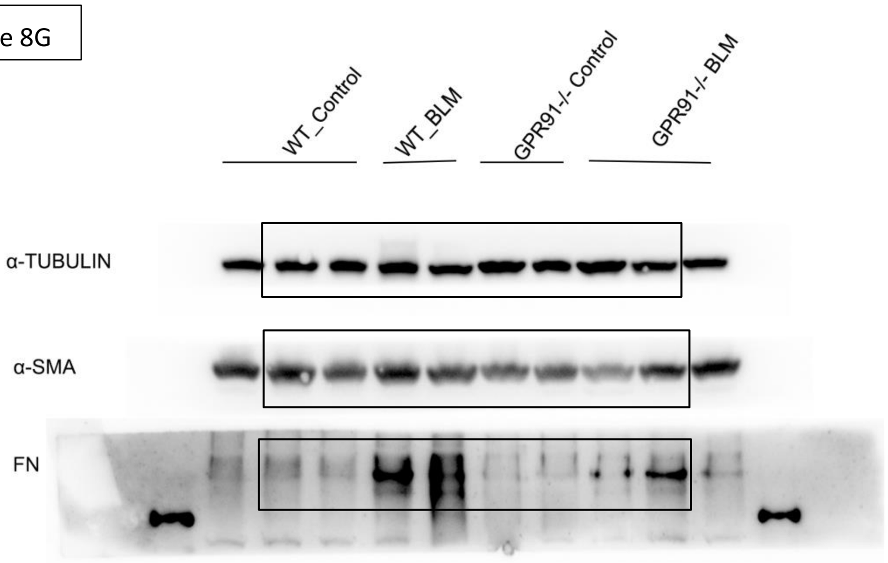

Figure 9A

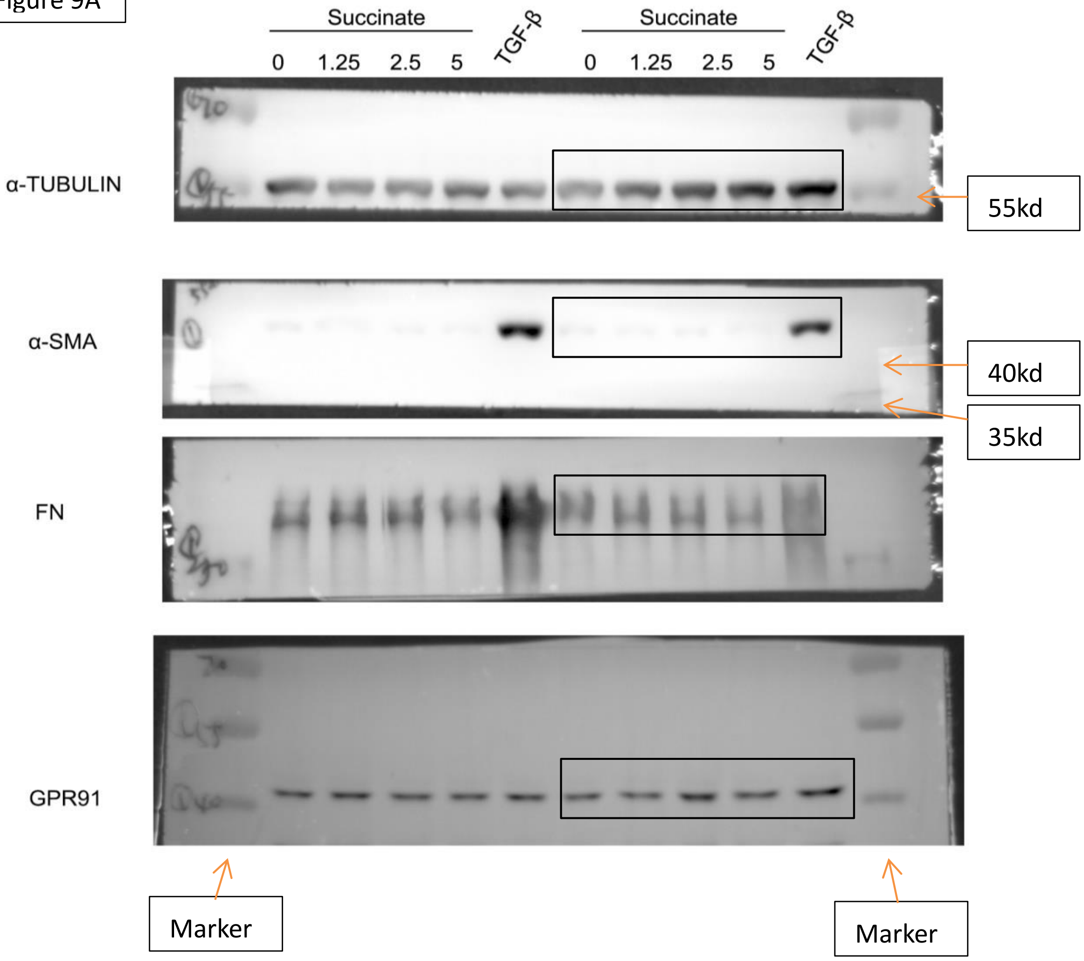

Figure 9A

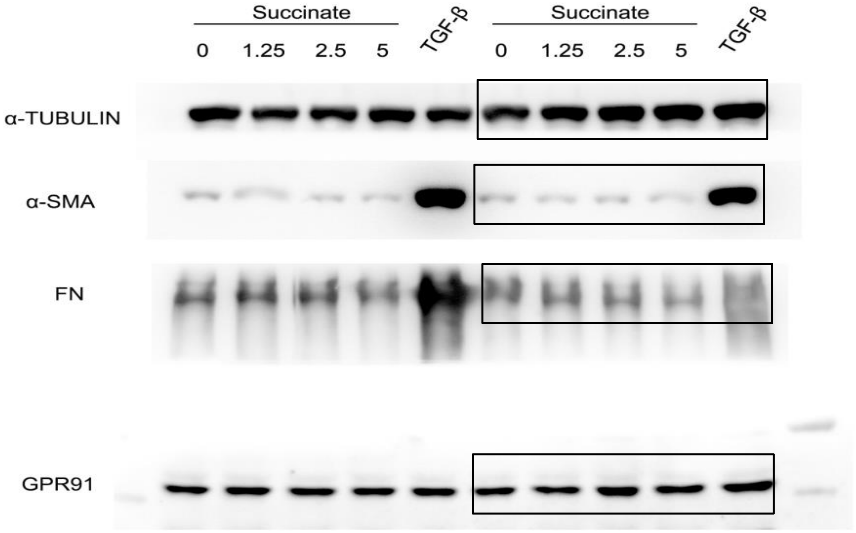

Figure 9E

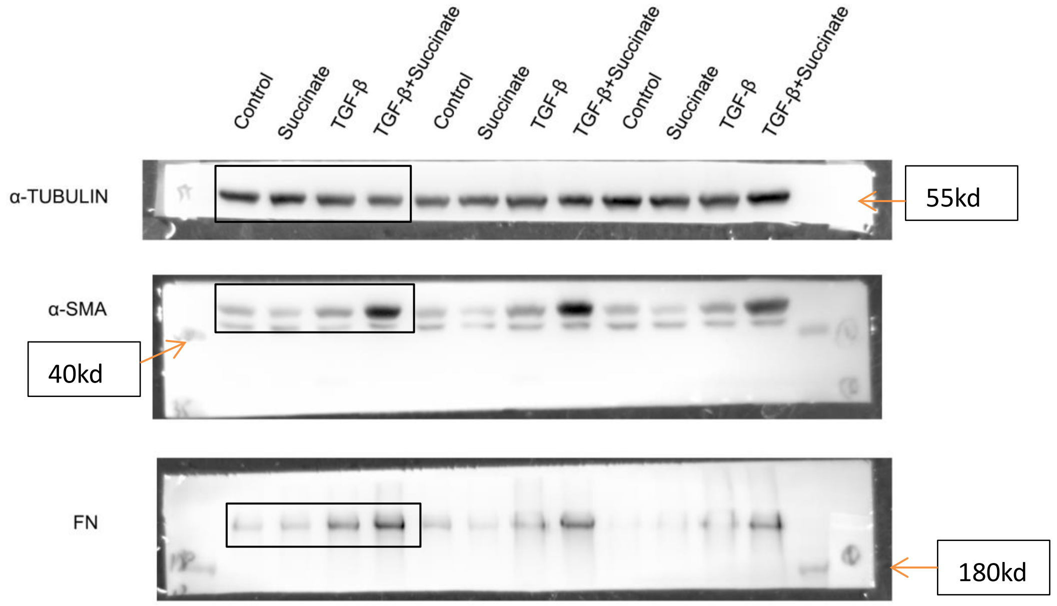

Figure 9E

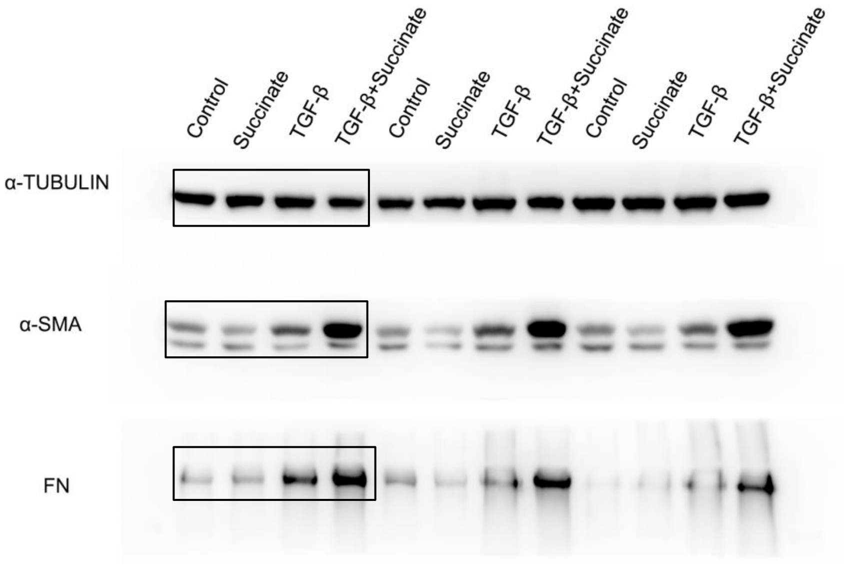

Figure 9H

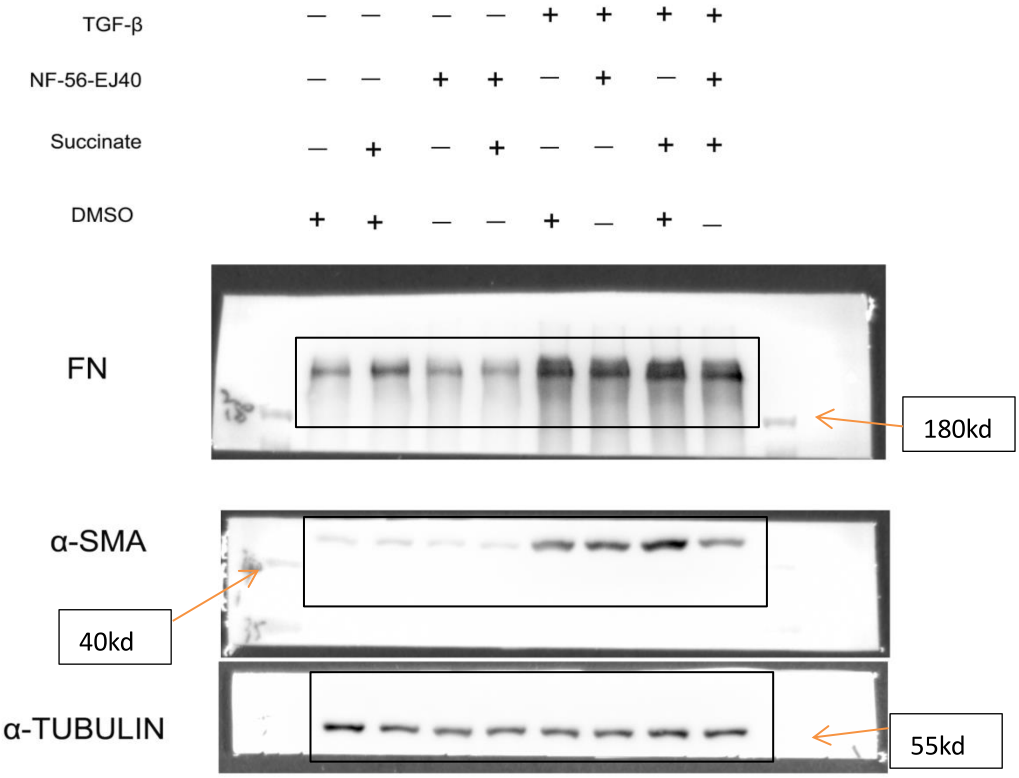

Figure 9H

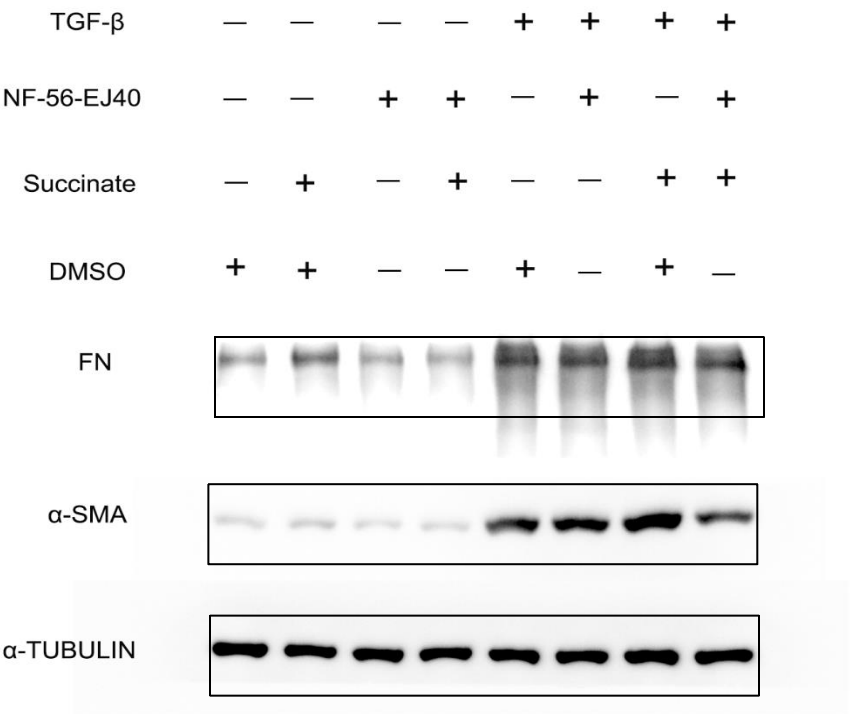

Figure10A

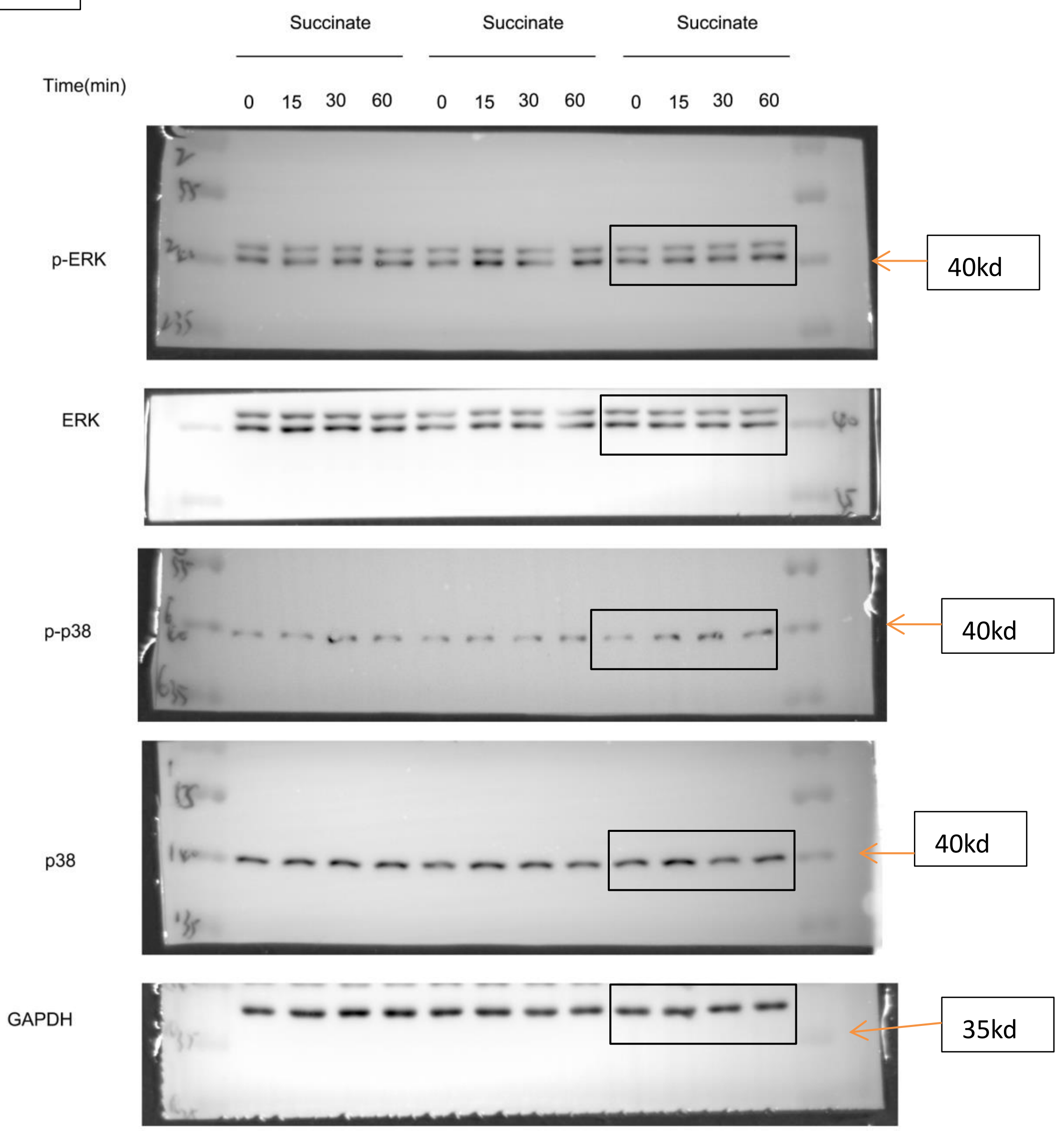
Succinate
Succinate
Succinate
Time(min)
0 15 30 60 0 15 30 60 0 15 30 60
p-ERK
40kd
ERK
p-p38
40kd
p38
40kd
GAPDH
35kd

Figure 10A

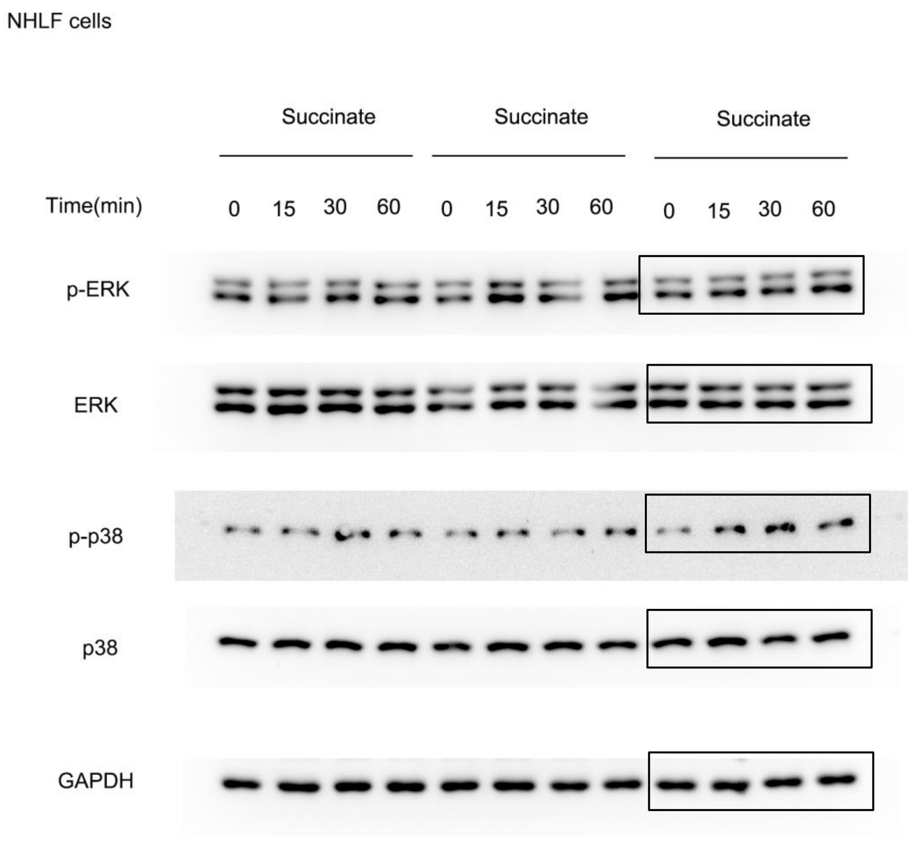

NHLF cells
Succinate
Succinate
Succinate
Time(min)
0 15 30 60 0 15 30 60 0 15 30 60
p-ERK
ERK
p-p38
p38
GAPDH

Figure 10D

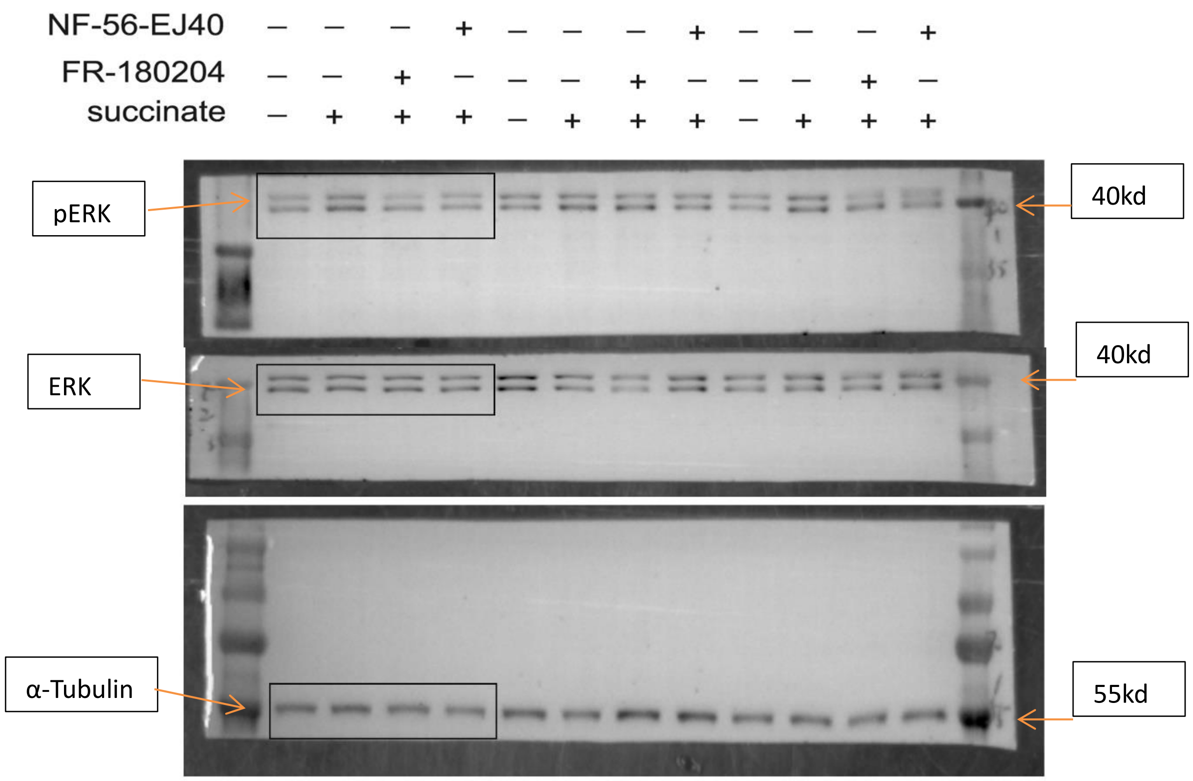
NF-56-EJ40 – – – + – – – + – – – +
FR-180204 – – + – – – + – – – + –
succinate – + + + – + + + – + + +
pERK
ERK
α-Tubulin
40kd
40kd
55kd

Figure 10F

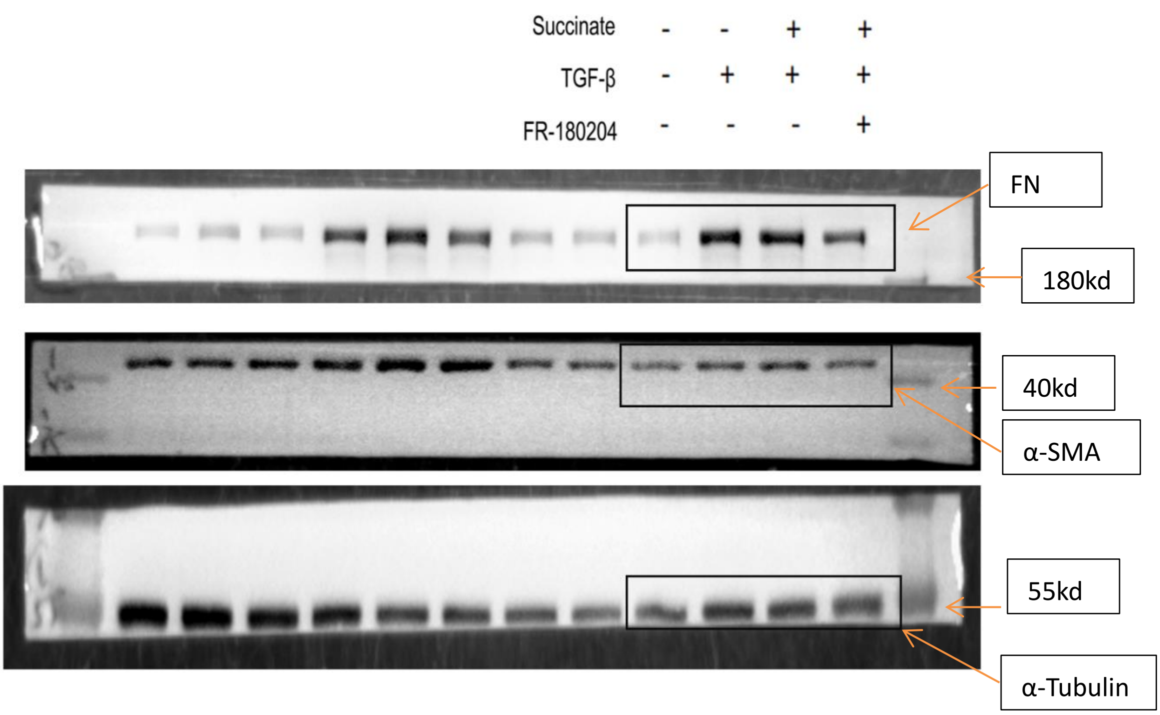

Supplemental Figure 3C

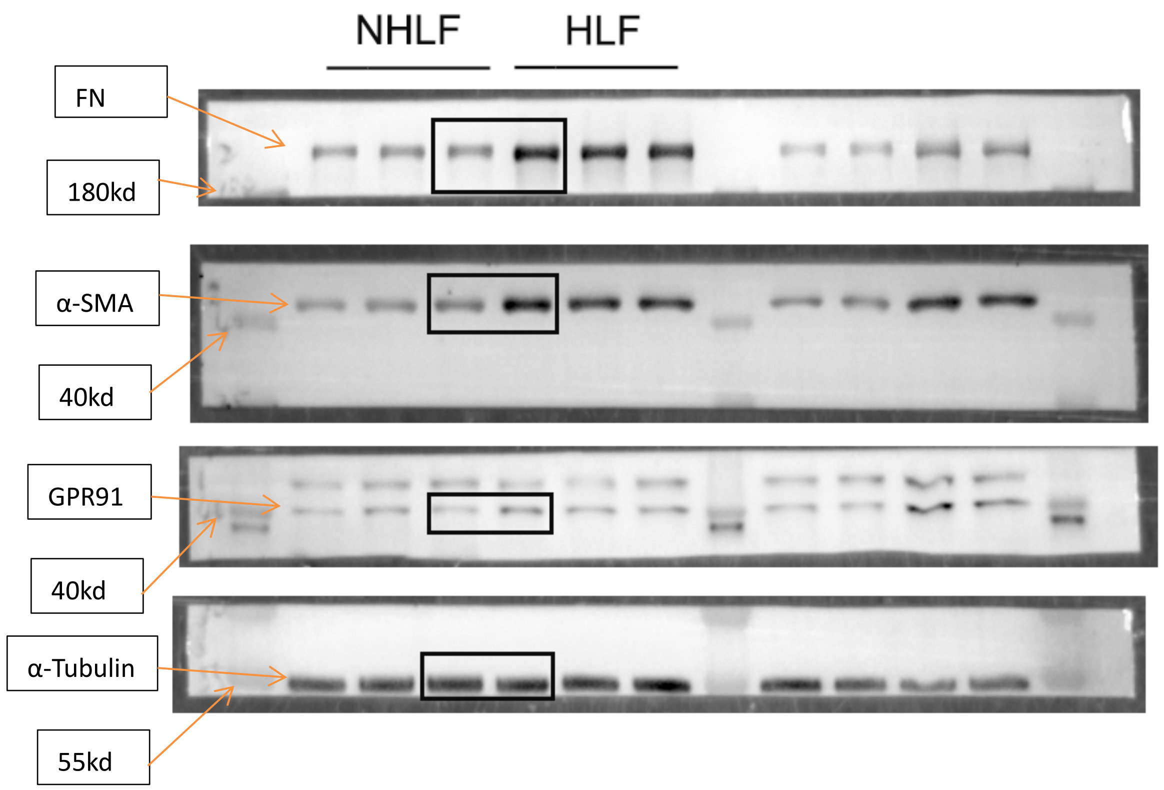

Supplemental Figure 4D

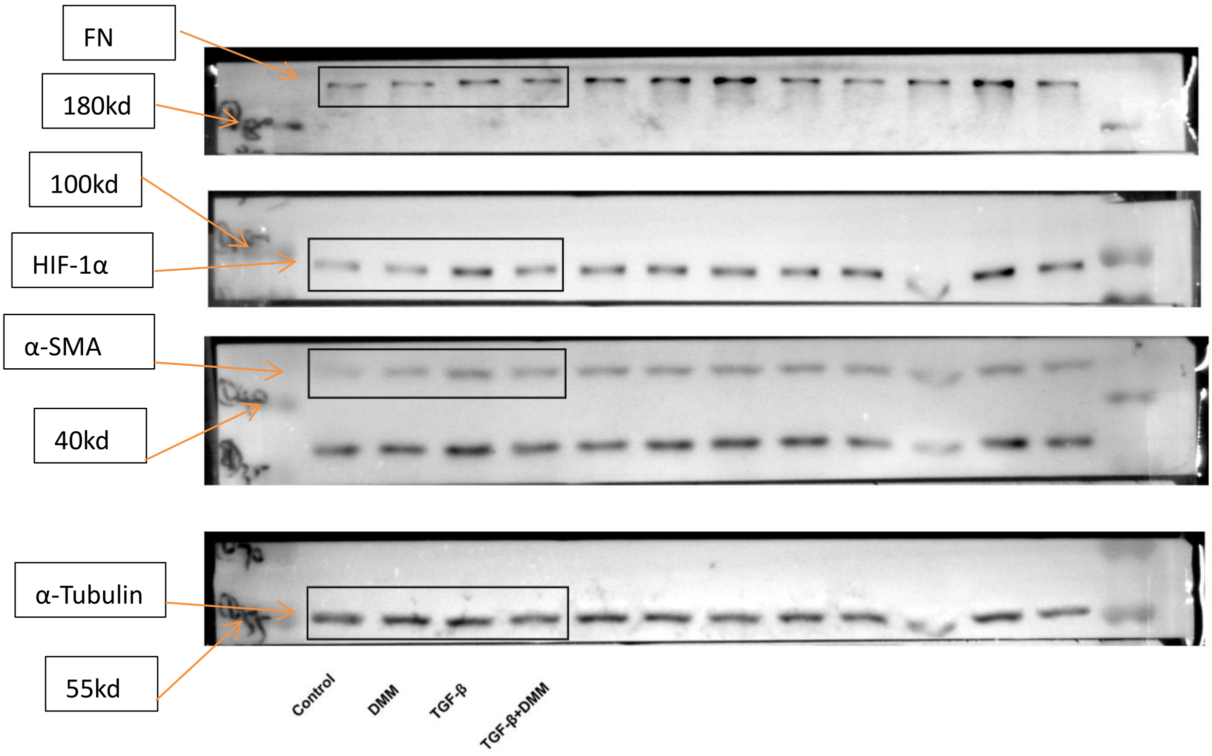

Supplement: Supplementary file 3 — Supplementary Information. [file 41598_2024_64844_MOESM3_ESM.pdf]
